# A Promiscuous Halogenase for the Derivatization of Flavonoids

**DOI:** 10.3390/molecules26206220

**Published:** 2021-10-14

**Authors:** Dominik Kolling, Marc Stierhof, Constanze Lasch, Maksym Myronovskyi, Andriy Luzhetskyy

**Affiliations:** 1 Department of Pharmaceutical Biotechnology, Saarland University, 66123 Saarbruecken, Germany; kollingdominik@gmail.com (D.K.); m.stierhof@t-online.de (M.S.); constanze.lasch@uni-saarland.de (C.L.); maksym.myronovskyi@uni-saarland.de (M.M.); 2 AMEG Department, Helmholtz Institute for Pharmaceutical Research Saarland, 66123 Saarbruecken, Germany

**Keywords:** halogenase, flavonoid, heterologous expression, *Frankia alni*, *Streptomyces albus* Del14

## Abstract

Halogenation often improves the bioactive properties of natural products and is used in pharmaceutical research for the generation of new potential drug leads. High regio- and stereospecificity, simple reaction conditions and straightforward downstream processing are the main advantages of halogenation using enzymatic biocatalysts compared to chemical synthetic approaches. The identification of new promiscuous halogenases for the modification of various natural products is of great interest in modern drug discovery. In this paper, we report the identification of a new promiscuous FAD-dependent halogenase, DklH, from *Frankia alni* ACN14a. The identified halogenase readily modifies various flavonoid compounds, including those with well-studied biological activities. This halogenase has been demonstrated to modify not only flavones and isoflavones, but also flavonols, flavanones and flavanonols. The structural requirements for DklH substrate recognition were determined using a feeding approach. The homology model of DklH and the mechanism of substrate recognition are also proposed in this paper.

## 1. Introduction

Halogenated natural products represent an important group of secondary metabolites that are predominantly produced by bacteria and fungi. Many of these compounds display relevant antibacterial activities, such as the clinically used antibiotics vancomycin, clindamycin, chloramphenicol and chlortetracycline [1,2,3,4]. Additionally, certain halogen-containing natural products, such as rebeccamycin, are well known for their antitumour effects [5]. In recent decades, halogenated secondary metabolites have gained increasing attention in drug discovery and medicinal chemistry due to their interesting pharmacological profile [6]. The introduction of halogens into the structures of natural products or synthetic compounds often improves their biological activity and physicochemical properties [7]. The electronic and lipophilic parameters of potential drugs are strongly affected by halogen substituents, often resulting in increased bioactivity [8,9,10]. For example, clinical trials of the flavonoid-derived inhibitor of cyclin-dependent kinase 2 (CDK2) flavopiridol have demonstrated its strong therapeutic potential to treat several forms of leukaemia [11]. The chemical structure of this anticancer agent is characterized by the presence of one chlorine atom attached to the aromatic moiety. This structural feature led to an increase in kinase inhibition by a factor of six compared to the flavopiridol derivative without the chlorine [6]. Crystallographic studies revealed that the alteration of the physiochemical properties caused by the chlorine strongly improved the binding of flavopiridol to CDK2 [12]. Moreover, in recent decades, it has been reported that halogen atoms have the ability to form directional noncovalent interactions in protein-ligand complexes [13]. This interesting binding mode was observed for the antimicrobial diphenyl ether triclosan (TCL) [14]. One of the three chlorine atoms present in this inhibitor molecule is directly involved in binding to the target protein trans-2-enoyl-ACP reductase (FabI) [15]. Due to this increased bioactivity, TCL is clinically used for the treatment of skin infections caused by methicillin-resistant *Staphylococcus aureus* (MRSA) [16].

The incorporation of halogens into the structures of drug leads using chemical approaches is associated with high effort and is often performed under rough reaction conditions [17,18]. Synthetic halogenation often requires additional reaction steps, such as the incorporation and subsequent removal of protecting groups [19]. Moreover, a complex mixture of reaction products is often obtained because of incomplete regioselectivity. To circumvent these problems, the development of enzymatic biocatalysts for the derivatization of natural products is of particular interest [20,21]. The utilization of enzymes is associated with a variety of favourable properties. Enzymatic reactions are characterized by their high regioselectivity and are performed in aqueous solution [22]. Enzymatic conversion is generally a one-step reaction. The enzymes can be immobilized and easily removed from the reaction matrix, which facilitates purification of the reaction products. Due to their beneficial properties such as more sustainable, environmentally friendliness and more cost-efficiency, the utilization of enzymatic catalysts instead of synthetic halogenation contributes greatly to green chemistry [23,24].

Most of the halogenases involved in the biosynthesis of natural products belong to the family of flavin-dependent oxidoreductases [25]. These enzymes use salts present in aqueous solutions as halogen sources. During catalysis, the halides chloride or bromide are enzymatically converted in the corresponding active intermediate hypohalous acid and then transferred to the substrate via a conserved lysine residue [26]. Common substrates of halogenases are aromatic compounds such as derivatives of tryptophan or phenylalanine [27]. The well characterized halogenase RebH is for example responsible for the regiospecific halogenation of tryptophan precursor in the biosynthesis of the natural compound rebeccamycin [28]. It has been further demonstrated for the fungal flavin-dependent halogenase RadH that several phenolic compounds were accepted as substrates and the relaxed substrate specificity allowed the construction of artificial biosynthetic pathways [29]. Of great interest is therefore the identification of other promiscuous halogenases for the modification of a variety of natural products. For instance, the halogenation of flavonoids, as demonstrated by flavopiridol, is a promising approach to generate new drug leads. Flavonoids are polyphenolic compounds that are widespread in the plant kingdom and well known for their interesting polypharmacological properties [30]. A variety of antioxidant, anti-inflammatory, anticancer, antibacterial and antiviral effects have been observed for this group of secondary metabolites [31,32,33,34]. Structurally, flavonoids consist of two aromatic benzene rings (A and B), which are connected by an oxygen containing C ring [35]. The presence of aromatic moieties within the flavonoid structure makes them potential substrates for flavin-dependent halogenases.

**Figure 1:**
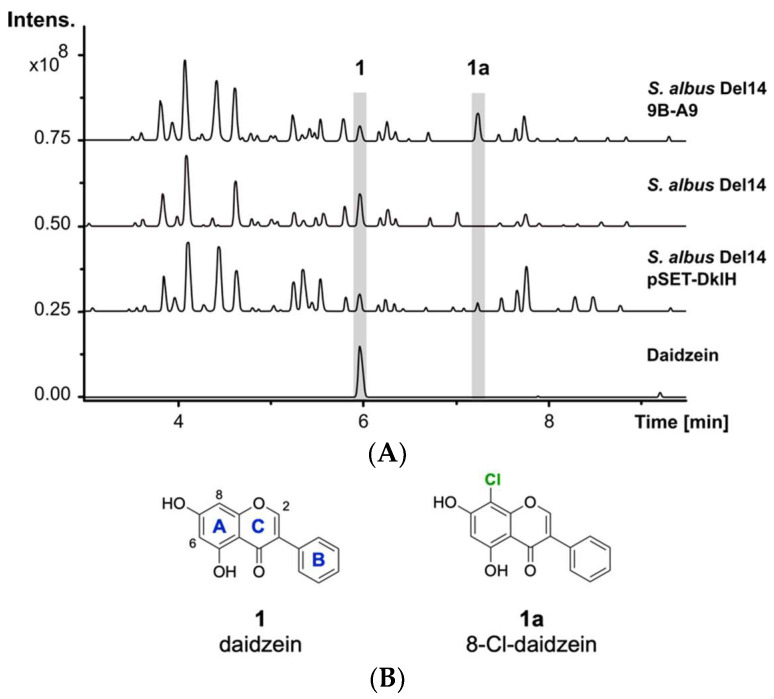
LC-MS detection of **1a**. (**A**) Base peak chromatograms of the crude extracts from *S. albus* 9B-A9, control strain *S. albus* Del14 and *S. albus* pSET-DklH and pure compound **1**. The peaks with retention times of 6.0 min and 7.2 min which are marked in grey correspond to **1** and **1a**. (**B**) Chemical structures of compounds **1** and **1a** (the flavonoid ring nomenclature is highlighted in blue and the chlorine atom is colored in green).

**Figure 2:**
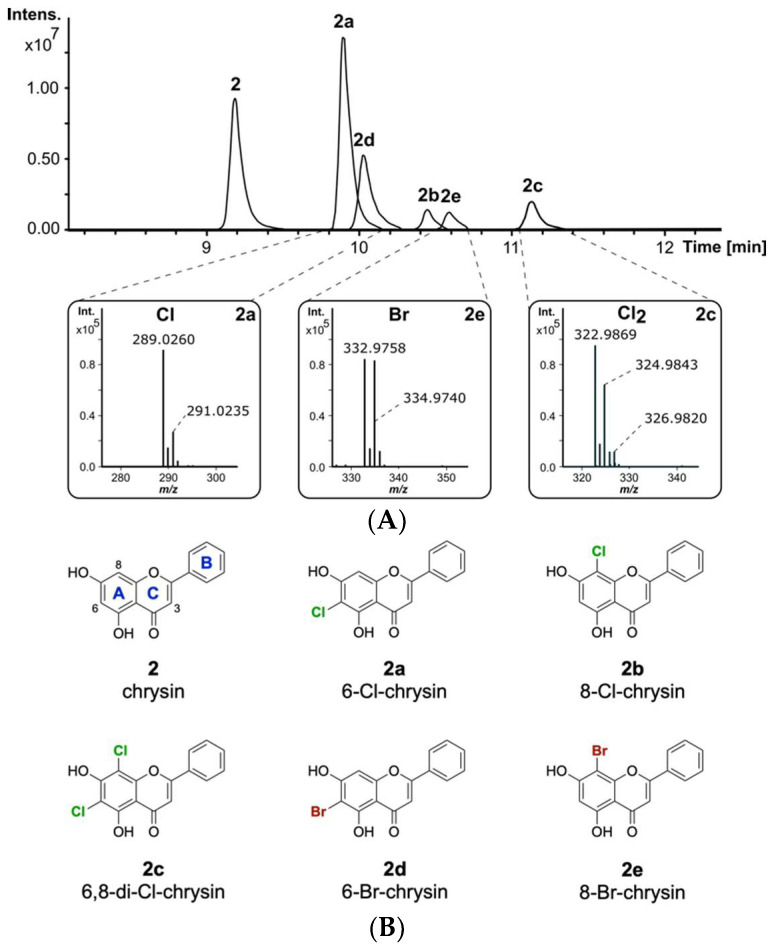
(**A**) Overlaid extracted ion chromatograms of the crude extracts from *S. albus* 9B-A9 supplemented with **2** and NaCl or NaBr. The characteristic isotope patterns of the halogenated products are shown. (**B**) The structures of the obtained halogenated derivatives of compound **2**. The structures of compounds (6-Cl-chrysin, 8-Cl-chrysin and 6,8-di-Cl-chrysin) **2a**–**2c** were confirmed by NMR, while the structures of **2d** and **2e** were predicted based on the LC-MS analysis results, the structure of chlorinated compounds and the proposed catalytic mechanism of the halogenase DklH (the flavonoid ring nomenclature and chlorine atoms are highlighted as described before, bromine atoms are colored in dark red).

In this work, we report the identification and characterization of the promiscuous halogenase DklH from *Frankia alni* ACN14a as a biosynthetic tool for the selective derivatization of flavonoids. A variety of flavonoid subclasses, such as flavones, isoflavones, flavonols, flavanones and flavanonols, were demonstrated to be accepted as substrates. For the two substrates daidzein **1** (Figure 1B) and chrysin **2** (Figure 2B), positions 6 and 8 of the aromatic A ring were determined to be the favourable positions for halogenation. The halogenase DklH is able to use both chloride and bromide to modify flavonoids, although chloride is preferred. Different substrate features required for the catalytic activity were determined by using a feeding approach. Further structural insights into the catalytic mechanism of DklH and the results of bioinformatic analysis results are also presented in this paper.

## 2. Results and Discussion

### 2.1. Identification of the Halogenase DklH through Heterologous Expression

The previously reported heterologous expression of the fosmid 9B-A9 from the genomic library of *F. alni* strain ACN14a led to the discovery of the compound fralnimycin [36]. Detailed LC-MS analysis of the crude extract from the recombinant strain *Streptomyces albus* 9B-A9 revealed a new peak, which was not present in the negative control strain without this cluster. High-resolution LC-MS analysis indicated that the identified peak corresponded to the compound with an [M + H]^+^ of 289.026 *m*/*z*. The following search in a natural product database uncovered the identity of the new peak as chlorinated **1** (Figure 1A) [37]. The isoflavone **1** is mainly found in soybeans and is also present in soy-based DNPM medium, which has been used for the cultivation of the *S. albus* strains [38]. The presence of **1** in DNPM medium was confirmed by the comparison of the retention time and exatct mass with a commercial standard (Santa Cruz Biotechnology, Inc., Dallas, TX, USA) using LC-MS analysis (t_R_ = 6.0 min, [M + H]^+^ of 255.066 *m*/*z*). To verify the identity of the analysed compound as the chlorine-containing derivative **1a**, the strain *S. albus* 9B-A9 was cultured on a preparative scale in DNPM medium. The culture broth was extracted with an equal amount of ethyl acetate, and the compound was purified by using size-exclusion and reversed-phase chromatographies. To investigate the position of chlorination, purified compound **1a** was used for NMR measurements. Analysis of the ^1^H-NMR spectrum confirmed the identity of the isolated product **1a** as 8-Cl-daidzein (Figure 1B).

The production of **1a** by the strain *S. albus* 9B-A9 implies that the corresponding halogenase is encoded by one of the genes within fosmid 9B-A9. To identify the halogenase gene, sequence analysis of fosmid 9B-A9 was performed. A gene annotated as a putative tryptophan-7-halogenase (FRAAL4634) was identified in the fosmid [39,40]. The encoded halogenase showed homology to NAD(P)/FAD-dependent oxidoreductases, which are often involved in the halogenation of aromatic compounds. The identified gene was named *dklH*. To suggest whether the identified gene is responsible for the modification of **1**, the gene was amplified by PCR and cloned into the expression vector pSET152 under the control of a strong TS81 promoter [41]. The obtained pSET-DklH construct was transferred into *S. albus* Del14, and the exconjugants were cultured in DNPM medium. LC-MS analysis of the crude extracts confirmed the production of **1a** by the strain *S. albus* Del14 pSET-DklH (Figure 1). This result unambiguously proves the involvement of the halogenase DklH in the modification of **1**.

### 2.2. Halogenation Pattern of the Flavone Chrysin

The activity of the halogenase DklH was originally detected in complex DNPM medium, which contains isoflavonoid **1**. To characterize the enzymatic properties of this halogenase, the cultivation conditions in minimal medium that allow feeding experiments were established. For this purpose, the strain *S. albus* 9B-A9 was cultivated in liquid minimal medium that was free of soya-derived flavonoids. To detect the activity of DklH, the medium was supplemented with the substrate **1** at a final concentration of 0.02 g/L. Additionally, the medium was supplemented with NaCl at a concentration of 1 g/L as a halogen source to increase halogenation efficiency. The strain *S. albus* Del14 was used as a negative control. LC-MS analysis revealed the successful conversion of compound **1** into **1a** by *S. albus* 9B-A9. Compound **1a** was not detected in the extract of *S. albus* Del14. The obtained results indicate that DklH was expressed and was active in minimal medium. The established cultivation conditions allowed the activity of DklH to be tested on substrates different than isoflavonoid **1**.

The naturally occurring compound **2** is one of the most interesting flavonoids produced in the plant kingdom. Compound **2** has been reported to display favourable pharmacological properties, such as inhibition of prostaglandin E2 production, which is known to be an important inflammatory mediator [42,43]. Additionally, anticancer effects by binding to the tumour-associated human protein kinase CK2 have been reported for flavone **2** [44]. Since halogenation of naturally occurring flavonoids was previously reported to increase their biological activity, it is of interest to develop methods for the derivatization of **2** [45]. To evaluate whether **2** will be modified by the halogenase DklH, this compound was fed to the culture of the strain *S. albus* 9B-A9 in minimal medium supplemented with NaCl. The strain *S. albus* Del14, without the fosmid, was used as a negative control. High-resolution LC-MS analysis of the culture extracts revealed the presence of three new halogenation products of **2** compared to the control (Figure 2A). The peaks with retention times of t_R_ = 9.9 min and t_R_ = 10.4 min had the same *m*/*z* of 289.026 [M + H]^+^, indicating that two different monochlorinated regioisomers of **2** were produced. The monochlorinated derivative **2a** with a shorter retention time was present in a greater amount. This suggests that the halogenase DklH may prefer one position over the other, leading to the production of the main chlorination product **2a** and of the side product **2b**. Interestingly, the third peak with retention time t_R_ = 11.1 min showed a mass difference of 68 *m*/*z* compared to the unmodified compound, which implied dichlorination. The presence of the characteristic mass shifts +2 *m*/*z* and +4 *m*/*z* in the mass spectrum of chrysin derivative **2c** with an [M + H]^+^ of 322.987 *m*/*z* further confirmed the presence of two chlorine atoms in its structure (Figure 2A). The low peak intensity of **2c** compared to the monochlorinated derivatives **2a** and **2b** indicated that dichlorination by DklH occurs with lower efficiency.

The ability of halogenases to use both chloride and bromide as halogen sources to form halogenated natural products has been previously reported [46]. The introduction of bromine often expands the biological activity and could be used for synthetic chemical derivatization [47]. The enzymatically obtained brominated compounds are then used as building blocks for further derivatization via cross-coupling reactions to expand the chemical diversity [48]. To evaluate whether the halogenase DklH accepts bromide in addition to chloride, the culture of the strain *S. albus* 9B-A9 in minimal medium was supplemented with compound **2** and NaBr instead of NaCl. LC-MS analysis of the crude extracts revealed the production of two new derivatives of **2**, **2d** and **2e**, with retention times of t_R_ = 10.0 min and t_R_ = 10.6 min. For both compounds **2d** and **2e**, the corresponding [M + H]^+^ peaks were identified as 332.976 *m*/*z*, with a characteristic mass difference of 78 *m*/*z* compared to unmodified flavonoid **2** (Figure 2A). Together with the results of MS analysis, the obtained data indicated that the detected compounds **2d** and **2e** correspond to two regioisomers of **2** containing one bromine at two different positions. Similar to the monochlorinated compounds, brominated derivative **2d** with a shorter retention time was present in a lower amount than the derivative with a longer retention time. In contrast to chlorination, in the bromination experiment, no derivative containing two bromine moieties was detected. In general, the absolute intensities of the peaks corresponding to the brominated compounds were significantly lower than those of the peaks of the chlorinated products. The reduced bromination efficiency of DklH compared to chlorination may explain why no dibrominated derivatives were observed.

Feeding experiments demonstrated that flavone **2** is modified by DklH at two different positions. Halogenation products modified either at one of the available positions or simultaneously at both positions were detected. To identify the positions at which compound **2** is halogenated, we set out to purify the halogenated products for subsequent NMR analysis. For this purpose, the strain *S. albus* 9B-A9 was cultured in minimal medium on a preparative scale and supplemented with either chloride or bromide. Compound **2** was fed to the culture at the time of inoculation at a concentration of 10 mg/L. Isolation of the brominated derivatives was not feasible due to the low production. The chlorinated compounds were purified using normal-phase and size-exclusion chromatographies. After the purification steps, two fractions were obtained, one containing a mixture of both monochlorinated derivatives **2a** and **2b** and the other containing the unmodified compound **2** together with the dichlorinated derivative **2c**. The purified fractions were used for NMR analysis.

Due to the small number of proton signals, the structures of the derivatives were mainly accomplished by analysing the HMBC correlations (Figures S29 and S35). The ^1^H-NMR spectrum of the first fraction showed the expected peak pattern for the monochlorinated compound **2a**, including the aromatic protons of the phenyl ring at δ_H_ 8.08 (2H), 7.58 (2H), 7.62 (1H), a singlet at δ_H_ 7.04 (CH-3) and a singlet corresponding to CH-6 or CH-8 (Figure S26). The similar chemical environments of CH-6 and CH-8 prevented precise annotation of the chlorine atom through 2D NMR experiments. Therefore, the exact position of the chlorine was determined by comparison of the carbon shift signals of the measured compound and the predicted ^13^C signals from ACD Labs (Table S3). The carbon and proton shifts of 6-Cl-chrysin were calculated at δ_C_ 103.6 (C-6) and δ_C,H_ 95.6, 6.70 (CH-8), precisely matching the observed shifts of δ_C_ 103.4 (C-6) and δ_C,H_ 94.8, 6.72 (CH-8). Therefore, monochlorinated product **2a** was identified as 6-Cl-chrysin. By carefully analysing the remaining proton signals in the ^1^H-NMR spectrum and the corresponding HMBC correlations, the presence of **2b** was also found in very small quantities (Figure S26). A full assignment could not be achieved due to the lack of correlations and low signal strength. However, the observed peaks at δ_H_ 6.41 (CH-6) and δ_C_ 98.3 (C-8) were closely related to the calculated 8-Cl-chrysin peaks at δ_H_ 6.61 (CH-6) and δ_C_ 100.5 (C-8), hence suggesting **2b** as the 8-Cl-chrysin side product (Table S3). These results demonstrate, that the halogenase DklH acts at positions 6 and 8 of the flavonoid scaffold (Figure 2B).

The ^1^H-NMR spectrum and HMBC correlations of the second purified fraction showed signals for precursor **2** and dichlorinated product **2c** in a ratio of approximately 3/2 (Figure S31). Comparison to the predicted ^13^C shift data of 6,8-Cl-chrysin obtained from ACD Labs further supported the assignment of the dichlorinated derivative **2c**, revealing halogenation at positions 6 and 8 (Table S4).

Considering that the mechanism of the reactions catalysed by the halogenase DklH is not altered by the nature of the halide used, it can be suggested that bromination of **2** also takes place at positions 6 and 8. Therefore, it was determined with high probability that brominated derivatives **2d** and **2e** correspond to 6-Br-chrysin and 8-Br-chrysin, respectively.

### 2.3. Analysis of the Substrate Specificity of the Halogenase DklH

**Figure 3:**
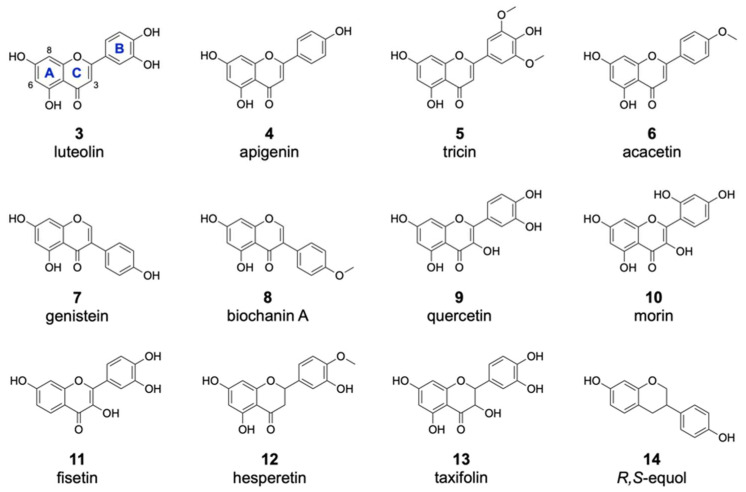
Substrates accepted by the halogenase DklH. The flavonoid substrates are listed according to structural groups: flavones (**3**–**6**), isoflavones (**7**,**8**), flavonols (**9**,**10**,**11**), a flavanone (**12**), a flavanonol (**13**) and an flavandiol (**14**).

The successful modification of **1** and **2** by DklH indicated that the studied halogenase is able to accept different flavonoid groups as substrates. Since compound **1** belongs to the isoflavone family and compound **2** belongs to the flavone family of natural products, it would be interesting to determine the structural requirements for the activity of DklH. For this purpose, a set of 35 flavonoids (Figures S1–S3) and compounds structurally similar to flavonoids were selected to study the substrate specificity of the halogenase DklH. These compounds were fed into the culture of strain *S. albus* 9B-A9 and *S. albus* Del14 pSET-DklH as described before, and the halogenation products were assayed using LC-MS (Figure 3).

Of the 16 members of the flavone group, enzymatic halogenation was only observed for luteolin **3**, apigenin **4**, tricin **5** and acacetin **6** (Table 1). For most of these substrates, the mono- and dichlorinated products were identified. The previously observed halogenation pattern of **2** implied with high probability that these other flavones are also modified at positions 6 and 8. In contrast to **2**, only one of two possible monochlorinated derivatives was detected for the flavones **3**–**6**. This result could be explained by the presence of additional hydroxyl moieties attached to the flavonoid B ring in these compounds, which clearly affected the chlorination pattern. In the case of the dichlorinated derivatives, we assumed that halogenation took place at both positions 6 and 8, as in the case of **2**. Brominated derivatives were also detected for the compounds **3**–**6**. However, these products were found in much lower amounts than the chlorinated derivatives. This is in accordance with the previously obtained data for **2** and further confirms that DklH prefers chloride to bromide. Interestingly, two different monobrominated derivatives were obtained for most of the compounds, indicating that bromination is apparently not influenced by the hydroxyl groups on the B ring.

**Figure 4:**
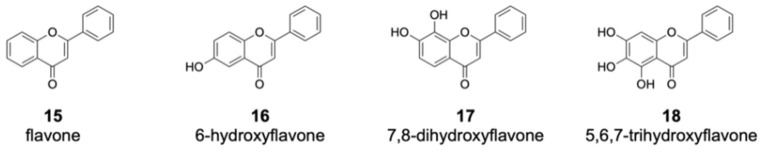
Members of the flavone group that were not enzymatically converted by DklH. The compounds **15** and **16** are characterized by the absence of a hydroxyl group at position 7. For the flavones **17** and **18**, one of the favourable halogenation positions, either 6 or 8, are substituted with a hydroxyl group.

Among the flavones fed into the culture medium, 12 compounds were not converted by DklH (Figures S2 and S3). Many of these unaccepted compounds (such as flavone **15** and 6-hydroxyflavone **16**) are characterized by the absence of a hydroxyl group at position 7 (Figure 4). 

This strongly indicates that the presence of a hydroxyl group at position 7 of the flavone scaffold is required for recognition by DklH. Halogenation was not observed in the case of 7,8-dihydroxyflavone **17** and 5,6,7-trihydroxyflavone **18**. Both of these compounds not only have the hydroxyl group at position 7 required for recognition by DklH but also have an additional hydroxyl group at either position 8 or 6. The presence of an additional hydroxyl group at one of two positions that should be halogenated clearly prevents the compounds from being converted by the halogenase.

From the group of isoflavones, three compounds were used for the feeding experiment (Table 1). As in the case of compound **1**, halogenated derivatives were observed for two other isoflavones, genistein **7** and biochanin A **8**. Interestingly, in contrast to **1**, two different monohalogenated and one dihalogenated derivatives were detected for both **7** and **8**. Moreover, two monobrominated derivatives of **8** were found. In general, the modification of both flavones and isoflavones clearly indicated that the halogenase DklH tolerates a shift of the B ring (Figure 3). This also indicated that the A and C ring core structure present in flavones and isoflavones might play a crucial role in their recognition as substrates.

For the flavonol group, 5 different compounds were analysed in the assay with halogenase DklH (Figure 3 and Figures S2 and S3). From the tested compounds, only quercetin (**9**), morin (**10**) and fisetin (**11**) were successfully halogenated, but each was halogenated only at one position. The relative efficiency of halogenation was quite low compared to the halogenation efficiencies of the flavones and isoflavones. No dichlorinated or brominated derivatives were detected in the analysed extracts. The group of flavonols is structurally characterized by the presence of a hydroxyl group at position 3, which is tolerated to some extent by the halogenase DklH but results in low turnover rates.

One representative from each of the three classes (flavanones, flavanonols and isoflavandiols) was analysed as a possible substrate of the halogenase DklH. Structurally, the tested compounds are characterized by a saturated bond between positions 2 and 3 and the presence of two stereochemical centres (Figure 3). For the flavanone hesperetin (**12**), two monochlorinated and a dichlorinated derivative were observed. Additionally, the corresponding bromine-containing derivative was detected, which indicates that DklH accepts **12** as a substrate. The flavanonol taxifolin (**13**) differs structurally from flavanones, as it contains an additional hydroxyl group at position 3. As in the case of the flavonols, the presence of this hydroxyl group in the structure of **13** strongly reduced the halogenation efficiency. Only one monochlorinated derivative of **13** was detected. The compound *R*,*S*-equol (**14**), which belongs to the isoflavandiols group, is characterized by the absence of a carbonyl moiety at position 4 and by a shift in of the aromatic B ring at position 3. These structural features of **14** were all tolerated by DlkH. Both monochlorinated and monobrominated derivatives of **14** were detected. However, no dichlorinated derivative of **14** was observed.

In summary, the substrate specificity of the halogenase DklH was determined by the feeding of various flavonoids. In general, it was demonstrated that the halogenase DklH acts on the aromatic A ring of the flavonoid scaffold. A hydroxyl group present at position 7 is required for DklH activity and it could be suggested that this important substituent directs halogenation. This enzyme shows certain promiscuity for the orientation of the flavonoid B ring, as both flavones and isoflavones were modified. Additionally, saturation of the bond between positions 2 and 3 is tolerated by DklH. Only the presence of a hydroxyl group at position 3 correlates with lower halogenation efficiency. The obtained results implied that the halogenase DklH can be used as a promising tool for the modification of various flavonoids.

### 2.4. Proposed Mechanism of DklH and Docking Analysis of the Substrate Chrysin

The feeding of different flavonoids provided some insights into the requirements for substrate recognition by the halogenase DklH. For this reason, it was interesting to characterize DklH in more detail and propose a suitable mechanism for halogenation based on the observed halogenation pattern. Initial sequence analysis revealed similarity between DklH and the group of tryptophan-halogenases. One of the most studied tryptophan-7-halogenase, RebH, is involved in the biosynthesis of the antitumour compound rebeccamycin [49,50]. This enzyme selectively catalyses chlorination of the tryptophan at position 7 of the aromatic ring. Due to the structural similarity between the flavonoid A ring and tryptophan, it was interesting to compare RebH with the halogenase DklH. To identify conserved regions and the functional sites of DklH, a bioinformatics analysis was performed. Multiple sequence alignment with RebH and the other halogenases, PrnA and CndH, revealed conserved residues that were previously described as flavin adenine dinucleotide (FAD) binding sites (Figure S4) [51]. This led to the assumption that DklH most likely belongs to the family of FAD-dependent halogenases.

**Figure 5:**
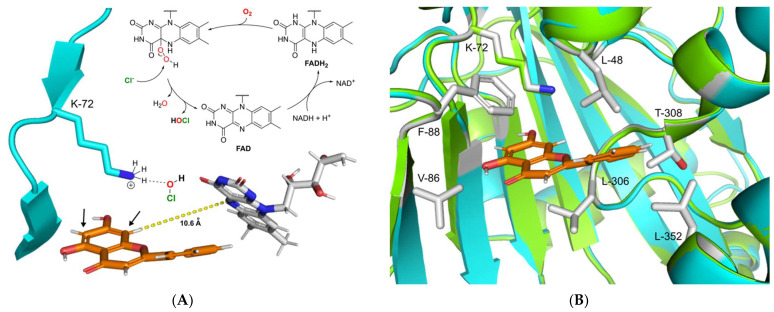
(**A**) Proposed catalytic mechanism of DklH. The halogenation agent hypochlorous acid is formed in an FAD-dependent manner and then coordinates through the active residue lysine-72 near the substrate binding pocket. Halogenation takes place at position 6 or 8 of substrate **2**, ortho to the 7-hydroxyl moiety. (**B**) Structural superimposition of the homology model of DklH (cyan) and template 3E1T (green). The ligand **2** is represented as orange sticks. Crucial residues responsible for substrate binding and cavity formation are shown as sticks coloured by element.

The catalytic mechanism of FAD-dependent halogenases has been an object of numerous studies. The highly reduced FADH_2_, which is required for the activity of these enzymes, is supplied by a cognate NAD-dependent flavin reductase. This coenzyme is directly involved in the formation of the halogenating agent hypohalous acid (HOX; X = Cl, Br) [52]. During this process FADH_2_ recruits molecular oxygen with the help of halogenase to form a peroxide-linked isoalloxazine intermediate (Figure 5A) [53]. This unstable species is then attacked by chloride or bromide, leading to the formation of hypohalous acid. The generated hypohalous acid is then transferred from the FAD binding site to the substrate binding cavity through a 10 Å long channel [54]. In the substrate pocket, hypohalous acid is coordinated by the highly conserved active site lysine residue, which then performs halogenation according to electrophilic aromatic substitution mechanism [25]. The electron-rich aromatic ring of the substrate attacks the electrophilic chlorine species, leading to halogen attachment and disruption of the aromatic system. The resulting intermediate known as the Wheland complex is characterized by a positive charge. In the case of RebH and other common FAD-dependent halogenases, this complex is stabilized by an interaction with aspartic or glutamic acid residues, which deprotonate the Wheland complex at the halogenated position [29,54]. This restores the aromaticity of the halogenated ring of the substrate to complete the reaction.

To gain insight into the possible catalytic mechanism of DklH, its 3D model was constructed with the help of the online server SWISSmodel [55]. The model was constructed based on the crystal structure of the chondrochloren halogenase CndH (PDB code: 3E1T), which showed the best coverage and evaluated based on TM-score (0.89 normalized by 473 residues) [56,57]. By carefully analysing the homology model of DklH, no aspartic or glutamic acid residues required for stabilization of the Wheland complex could be identified in the substrate binding pocket of the enzyme. This implied the DklH had a catalytic mechanism that was different from those of the other characterized FAD-dependent halogenases. 

We propose that the presence of the hydroxyl group is essential for DklH activity at position 7 of the flavonoid substrates compensates for the absence of these particular amino acid residues that coordinate to the Wheland complex. Partial deprotonation of the hydroxyl group of the flavonoids upon binding in the substrate pocket of DklH increases the electron density in the aromatic A ring. This facilitates electrophilic attachment of the chlorine at position 6 or 8 modulated by the lysine residue in the active site of DklH (position 72). The positive charge of the reaction intermediate is then stabilized by the electron-donating effect of the deprotonated hydroxyl group at position 7.

To more deeply analyse the substrate pocket of the halogenase DklH and the mechanism of substrate binding, a docking experiment of compound **2** into the homology model of DklH was performed by using MOE software [58]. The conserved lysine-72 present in the active site was used to define the docking parameters, and the most favourable binding pose was identified according to the observed interactions (Figure 5B). The binding pocket of DklH was found to be completely composed of nonpolar amino acid residues, which are tailored for correct orientation of the hydrophobic flavonoid substrates. Phenylalanine-88 seems to play a crucial role in substrate orientation by forming a π-stacking interaction with the A ring of **2**. Hydrophobic interactions were also observed between the B ring and leucine-48. Leucine-306 of the substrate binding pocket has been shown to further facilitate the correct binding of the C ring of **2**. This proposed model for the docking of **2** in the substrate pocket of DklH can be applied to other structurally related flavonoids.

## 3. Materials and Methods

### 3.1. General Experimental Procedures

The strains, fosmids and plasmids used in this study are listed in Table S1. *Escherichia coli* strains were cultured in lysogenic broth (LB) medium under standard conditions [59]. For conjugation and sporulation, the strain *S. albus* Del14 was grown on soya flour mannitol agar (MS agar) and in liquid tryptic soy broth (TSB; Sigma-Aldrich, St. Louis, MO, USA) at 28 °C. The production media DNPM (40 g/L dextrin, 21 g/L MOPS, 7.5 g/L soytone and 5 g/L baking yeast, pH 6.8) and minimal medium (0.5 g/L L-asparagine, 0.5 g/L K_2_HPO_4_, 0.2 g/L MgSO_4_·7 H_2_O, 0.01 g/L FeSO_4_·7 H_2_O, 10 g/L glucose, 2 g/L tryptone and 1 g/L NaX (X = Cl or Br), pH 7.0–7.2) were used for secondary metabolite expression. The antibiotics apramycin and nalidixic acid were added in appropriate concentrations as required.

### 3.2. DNA Isolation and Manipulation

Extraction of the fosmid from *F. alni* ACN14a constructed a genomic library, DNA manipulation, transformation into *E. coli* and intergeneric conjugation between *E. coli* and *S. albus* Del14 were performed according to standard protocols [60,61,62]. The halogenase gene *dklH* encoded by fosmid 9B-A9 was amplified with Phusion DNA polymerase (Thermo Fisher Scientific, Waltham, MA, USA) using the primers listed in Table S1. PCR products were purified from agarose gel with the QIAquick Gel Extraction Kit (Qiagen, Hilden, Germany). For heterologous expression the plasmid pSET152 and the strong TS81 promoter were used [41]. The plasmid was purified by using the BACMAX^TM^ DNA purification kit (Lucigen, Middleton, WI, USA). For cloning, the restriction enzymes provided by Thermo Fisher Scientific or New England BioLabs (Ipswich, MA, USA) and T4 DNA Ligase (Thermo Fisher Scientific) were applied according to supplier’s protocol. The obtained PCR fragment was digested with *Xba*I and *EcoR*V and cloned in the similarly linearized plasmid pSET152 to generate the construct pSET-DklH. The correct cloning procedure was confirmed by digestion and Sanger sequencing using the check primers listed in Table S1. Generated recombinant plasmids and fosmid 9B-A9 were introduced into *S. albus* Del14 and selected by acquired resistance.

### 3.3. Cultivation, Feeding Experiment and Metabolite Extraction

For the initial cultivation experiments with *S. albus* Del14, recombinant strains with fosmid 9B-A9 and pSET-DklH were grown in 15 mL of TSB for 1 day at 28 °C and 180 rpm. The next day, 100 mL of DNPM medium was inoculated with 1 mL of seed culture in a 500 mL baffled flask. The cultures were grown in an Infuros multitron shaker (Infuros AG, Basel, Switzerland) for 7 days under the same conditions described for the seed culture. Metabolite extraction from 15 mL of culture supernatant was carried out with an equal amount of ethyl acetate in Falcon tubes. After shaking for 1 h, the organic phase was separated by centrifugation and evaporated under continuous nitrogen flow overnight in a concentrator. The obtained dried crude extract was stored at 4 °C.

Flavonoid feeding experiments were performed in minimal medium. Fifteen millilitres of minimal seed culture of the same strains described above was incubated for 1 day at 28 °C and 180 rpm. One day later, 0.5 mL of preculture was used to inoculate 50 mL of DM in a 300 mL baffled flask. The respective flavonoids dissolved in DMSO (stock concentration 20 mg/mL) were added at a final concentration of 0.02 mg/mL. Cultures were grown for 7 days in the dark to avoid flavonoid degradation. For metabolite extraction, the standard procedure was performed.

For large-scale cultivation (10 L), the recombinant strain *S. albus* 9B-A9 was grown in 100 mL of DNPM in a 500 mL baffled flask. After 7 days, the metabolites were extracted from the supernatant with an equal amount of ethyl acetate. The crude extract was evaporated under reduced pressure and stored at 4 °C. For **2** feeding purposes, 10 L of minimal medium was used, and flavonoids were added at a final concentration of 10 mg/L. The downstream cultivation and extraction processes were performed as described above.

### 3.4. Mass Spectrometry (MS) and Metabolite Analysis

Dry crude extracts were dissolved in methanol prior to MS analysis. Sample compounds were separated on a Dionex UltiMate 3000 UPLC (Thermo Fisher Scientific) coupled to a PDA detector (stationary phase: 100 mm ACQUITY UPLC BEH C18 1.7 µm column (Waters Corporation, Milford, MA, USA), mobile phase: linear gradient of [A] ddH_2_O + 0.1% formic acid/[B] acetonitrile + 0.1% formic acid, 5% to 95% at flow rate 0.6 mL/min). Mass detection was carried out by coupling to an amaZon speed (Bruker, Billerica, MA, USA) or a MaXis II (Bruker) mass spectrometer, which provides a mass accuracy of 5 ppm. Spectra were aquired using positive ionization mode and with a mass selection range of *m*/*z* 100 to 2000. Compass Data Analysis 4.2 was used for mass spectrometry data analysis.

### 3.5. Purification

For the purification of **1a**, size-exclusion chromatography (SEC; stationary phase: Sephadex-LH 20; mobile phase: isocratic elution with 100% methanol) was used as the first attempt. Then, further purification was achieved by using semipreparative reversed-phase HPLC (Dionex UltiMate 3000, Thermo Fisher Scientific). The compounds were separated with a C18 column (Synergi 10 µm, 250 × 10 mm; Phenomenex, Aschaffenburg, Germany) with a mobile phase containing 0.1% formic acid solution in acetonitrile. Fractions containing pure **1a** were pooled, the solvent was evaporated under reduced pressure, and the final compound was analysed by NMR.

For the purification of **2a** and the other derivatives normal-phase chromatography was initially used. The crude extract was dry loaded onto Celite 545 (Merck KGaA, Darmstadt, Germany) as the stationary phase and filled in cartridges (Biotage, Uppsala, Sweden). The mobile phase consisted of hexane (solvent A), chloroform (solvent B), ethyl acetate (solvent C) and methanol (1:1:1:1). A linear gradient of each pair of solvents (A-B, B-C, and C-D) was used for separation. Fractions containing chlorinated **2** were identified by LC-MS analysis, pooled, dried under reduced pressure and redissolved in methanol. The second purification step was performed by using SEC (stationary phase: Sephadex-LH 20; mobile phase: isocratic elution with 100% methanol). The resulting compounds were analysed by LC-MS and pooled to obtain fraction 1 (a mixture of **2a** and **2b**) and fraction 2 (a mixture of **2** and **2c**).

### 3.6. Nuclear Magnetic Resonance (NMR) Spectroscopy

The NMR spectra of 8-Cl-daidzein (**1a**) 6-Cl-chrysin (**2a**), 8-Cl-chrysin (**2b**) and 6,8-di-Cl-chrysin (**2c**) were aquired on a Bruker Avance, UltraShield 500 MHz spectrometer (Bruker, BioSpin GmbH, Rheinstetten, Germany) equipped with a 5 mm BBO probe at 298 K. Chemical shifts (δ) are reported in parts per million (ppm) relative to TMS. Deuterated DMSO (DMSO-d_6_) (δ_H_ 2.50 ppm, δ_C_ 39.51 ppm) was used as the solvent. Edited-HSQC, HMBC and ^1^H-^1^H COSY spectra were recorded using the standard pulse programs from TOPSPIN v.3.6 software.

### 3.7. Bioinformatics Analysis and Homology Modelling

The genome of *Frankia alni* ACN14a was analysed by using Geneious 9.1.2 software [63]. The DNA sequence of DklH is available in GenBank under accession number Q0RGW0. The online server NCBI BLAST was used for sequence analysis and identification of related homologues [64]. Multiple sequence alignments were performed using the online tool T-coffee [65]. For the development of a homology model of DlkH, the SWISS-MODEL homology server was used [55]. The software identified chondrochloren halogenase (CndH) from *Chondromyces crocatus* Cm c5 (PDB code: 3E1T) as a suitable template (percent identity 37%, query cover 78%) to generate a 3D structure of DklH. Subsequently, the docking of **2** in the DklH model was carried out by using MOE 2019 software [58]. Active lysine-72 was selected to direct the docking position of **2** into the proposed substrate binding site of the DklH model. The relative position of FAD was elucidated by superimposition of the structure of CndH with the bound coenzyme and DklH. All structural data were visualized with the aid of PyMOL (Molecular Graphics System, Version 2.0 Schrödinger LLC, New York, NY, USA) [66].

## Figures and Tables

**Table 1:** Halogenation products observed by feeding experiments listed according to flavonoid subclass.

Subclass	Flavonoid	Halide	Product		Yield * [%]
Flavone	Chrysin (**2**)	Cl^−^	6-Chlorochrysin	(**2a**)	60.8
			8-Chlorochrysin	(**2b**)	4.0
			6,8-Dichlorochrysin	(**2c**)	10.3
		Br^−^	Bromochrysin	(**2d/e**)	27.1/3.7
	Luteolin (**3**)	Cl^−^	Chloroluteolin	(**3a**)	12.3
			Dichloroluteolin	(**3b**)	86.6
		Br^−^	Bromoluteolin	(**3c/d**)	1.0/7.4
	Apigenin (**4**)	Cl^−^	Chloroapigenin	(**4a**)	76.7
			Dichloroapigenin	(**4b**)	16.3
		Br^−^	Bromoapigenin	(**4c/d**)	2.5/21.1
	Tricin (**5**)	Cl^−^	Chlorotricin	(**5a**)	62.5
		Br^−^	Bromotricin	(**5b**)	78.8
	Acacetin (**6**)	Cl^−^	Chloroacacetin	(**6a**)	30.1
			Dichloroacacetin	(**6b**)	40.7
		Br^−^	Bromoacacetin	(**6c/d**)	9.8/n.d.
Isoflavone	Daidzein (**1**)	Cl^−^	8-Chlorodaidzein	(**1a**)	24.8
		Br^−^	Bromodaidzein	(**1b**)	39.7
	Genistein (**7**)	Cl^−^	Chlorogenistein	(**7a/b**)	5.9/52.2
			Dichlorogenistein	(**7c**)	28.3
		Br^−^	n.d.	n.d.	n.d.
	Biochanin A (**8**)	Cl^−^	Chlorobiochanin A	(**8a/b**)	5.9/22.3
			Dichlorobiochanin A	(**8c**)	25.8
		Br^−^	Bromobiochanin A	(**8d/e**)	3.7/5.1
Flavonol	Quercetin (**9**)	Cl^−^	Chloroquercetin	(**9a**)	35.0
		Br^−^	-	-	-
	Morin (**10**)	Cl^−^	Chloromorin	(**10a**)	72.2
		Br^−^	-	-	-
	Fisetin (**11**)	Cl^−^	Chlorofisetin	(**11a**)	48.2
		Br^−^	-	-	-
Flavanone	Hesperetin (**12**)	Cl^−^	Chlorohesperetin	(**12a/b**)	0.9/46.7
			Dichlorohesperetin	(**12c**)	43.3
		Br^−^	Bromohesperetin	(**12d**)	2.4/1.4
Flavanonol	Taxifolin (**13**)	Cl^−^	Chlorotaxifolin	(**13a**)	15.4
		Br^−^	-	-	-
Isoflavandiol	*R*,*S*-Equol (**14**)	Cl^−^	*R*,*S*-Chloroequol	(**14a/b**)	10.7/3.6
		Br^−^	*R*,*S*-Bromoequol	(**14c/d**)	14.9/10.0

* The yield was calculated by measuring the UV absorption at 280 nm.

## Data Availability

Not applicable.

## Supplementary Materials

The following are available online, Table S1: Bacterial strains, fosmids, plasmids and primers used in this work; Figure S1: Outline of DklH substrates classified by flavonoid substructure; Figure S2: Display of flavones and related derivatives not accepted by DklH; Figure S3: Display of compounds not converted by DklH; Figure S4: Multiple sequence alignment; Figure S5: Display of homology model of DklH; Figure S6: Extracted ion chromatogram of luteolin feeding experiment; Figure S7: Extracted ion chromatogram of apigenin feeding experiment; Figure S8: Extracted ion chromatogram of tricin feeding experiment; Figure S9: Extracted ion chromatogram of acacetin feeding experiment; Figure S10: Extracted ion chromatogram of daidzein feeding experiment; Figure S11: Extracted ion chromatogram of genistein feeding experiment; Figure S12: Extracted ion chromatogram of biochanin A feeding experiment; Figure S13: Extracted ion chromatogram of quercetin feeding experiment; Figure S14: Extracted ion chromatogram of morin feeding experiment; Figure S15: Extracted ion chromatogram of fisetin feeding experiment; Figure S16: Extracted ion chromatogram of hesperetin feeding experiment; Figure S17: Extracted ion chromatogram of taxifolin feeding experiment; Figure S18: Extracted ion chromatogram of *R*,*S*-equol feeding experiment; Table S2: LC-MS quantification and AUC determination for the chlorinated flavonoids; Table S3: Yield [%] determination for the chlorinated flavonoids based on the estimated AUC; Table S4: LC-MS quantification and AUC determination for the brominated flavonoids; Table S5: Yield [%] determination for the chlorinated flavonoids based on the estimated AUC; Table S6: ^13^C-NMR (125 MHz) and ^1^H-NMR (500 MHz) data of 8-Cl-daidzein and reference date from the literature; Figure S19: Structure of 8-Cl-daidzein; Figure S20: ^1^H-NMR spectrum of 8-Cl-daidzein (DMSO-d_6_, 500 MHz); Figure S21: ^13^C-NMR spectrum of 8-Cl-daidzein (DMSO-d_6_, 125 MHz); Figure S22: COSY spectrum of 8-Cl-daizein (DMSO-d_6_, 500 MHz); Figure S23: HSQC spectrum of 8-Cl-daidzein (DMSO-d_6_, 500 MHz); Figure S24: HMBC spectrum of 8-Cl-daidzein (DMSO-d_6_, 500 MHz); Table S7: ^13^C-NMR (125 MHz) and ^1^H-NMR (500 MHz) data of 6-Cl-chrysin **2a** and the minor side product 8-Cl-chrysin **2b** including their associated HOSE (Hierarchical Organization of Spherical Environments) code predictions from ACD; Figure S25: Structure of 6-Cl-chrysin and 8-Cl-chrysin; Figure S26: ^1^H-NMR spectrum showing the compounds A-D and the signals of protons 3, 6 and 8 of **2a**, **2b**, **2c** and the precursor **2** (DMSO-d_6_, 500 MHz); Figure S27: COSY spectrum of 6-Cl-chrysin including side products (DMSO-d_6_, 500 MHz); Figure S28: Edited-HSQC spectrum of 6-Cl-chrysin including side products (DMSO-d_6_, 500 MHz); Figure S29: HMBC spectrum of 6-Cl-chrysin including side products (DMSO-d_6_, 500 MHz); Table S8: ^13^C-NMR (125 MHz) and ^1^H-NMR (500 MHz) data of 6,8-Cl-chrysin including the associated HOSE (Hierarchical Organization of Spherical Environments) code predictions from ACD; Figure S30: Structure of 6,8-Cl-chrysin; Figure S31: ^1^H-NMR spectrum showing the compounds 6,8-Cl-chrysin (1c) and the precursor chrysin (1) including the signals of their protons 3, 6 and 8 (DMSO-d_6_, 500 MHz); Figure S32: ^13^C-NMR spectrum of 6,8-Cl-chrysin and the precursor chrysin (DMSO-d_6_, 125 MHz); Figure S33: COSY spectrum of 6,8-Cl-chrysin and the precursor chrysin (DMSO-d_6_, 500 MHz); Figure S34: Edited HSQC spectrum of 6,8-Cl-chrysin and the precursor chrysin (DMSO-d_6_, 500 MHz); Figure S35: HMBC spectrum of 6,8-Cl-chrysin and the precursor chrysin (DMSO-d_6_, 500 MHz).

## Sample Availability

Samples of the compounds are available from the authors.

## References

[1] Liu J., Volk K.J., Lee M.S., Pucci M., Handwerger S. (1994). Binding Studies of Vancomycin to the Cytoplasmic Peptidoglycan Precursors by Affinity Capillary Electrophoresis. Anal. Chem..

[2] Spížek J., Řezanka T. (2004). Lincomycin, Clindamycin and Their Applications. Appl. Microbiol. Biot..

[3] Sathya A., Prabhu T., Ramalingam S. (2020). Structural, Biological and Pharmaceutical Importance of Antibiotic Agent Chloramphenicol. Heliyon.

[4] Kasanah N., Triyanto T. (2019). Bioactivities of Halometabolites from Marine Actinobacteria. Biomolecules.

[5] Pommerehne K., Walisko J., Ebersbach A., Krull R. (2019). The Antitumor Antibiotic Rebeccamycin—Challenges and Advanced Approaches in Production Processes. Appl. Microbiol. Biot..

[6] Hernandes M.Z., Cavalcanti S.M.T., Moreira D.R.M., Junior W.F.d.A., Leite A.C.L. (2010). Halogen Atoms in the Modern Medicinal Chemistry: Hints for the Drug Design. Curr. Drug. Targets.

[7] Jiang S., Zhang L., Cui D., Yao Z., Gao B., Lin J., Wei D. (2016). The Important Role of Halogen Bond in Substrate Selectivity of Enzymatic Catalysis. Sci. Rep.-UK.

[8] Xu Z., Yang Z., Liu Y., Lu Y., Chen K., Zhu W. (2014). Halogen Bond: Its Role beyond Drug–Target Binding Affinity for Drug Discovery and Development. J. Chem. Inf. Model..

[9] Bernini R., Pasqualetti M., Provenzano G., Tempesta S. (2015). Ecofriendly Synthesis of Halogenated Flavonoids and Evaluation of Their Antifungal Activity. New J. Chem..

[10] Dias T.A., Duarte C.L., Lima C.F., Proença M.F., Pereira-Wilson C. (2013). Superior Anticancer Activity of Halogenated Chalcones and Flavonols over the Natural Flavonol Quercetin. Eur. J. Med. Chem..

[11] Senderowicz A.M. (1999). Flavopiridol: The First Cyclin-Dependent Kinase Inhibitor in Human Clinical Trials. Investig. New Drug.

[12] Deep A., Marwaha R.K., Marwaha M.G., Jyoti, Nandal R., Sharma A.K. (2018). Flavopiridol as Cyclin Dependent Kinase (CDK) Inhibitor: A Review. N. J. Chem..

[13] Mendez L., Henriquez G., Sirimulla S., Narayan M. (2017). Looking Back, Looking Forward at Halogen Bonding in Drug Discovery. Molecules.

[14] Cavallo G., Metrangolo P., Milani R., Pilati T., Priimagi A., Resnati G., Terraneo G. (2016). The Halogen Bond. Chem. Rev..

[15] Schiebel J., Chang A., Lu H., Baxter M.V., Tonge P.J., Kisker C. (2012). Staphylococcus Aureus FabI: Inhibition, Substrate Recognition, and Potential Implications for In Vivo Essentiality. Structure.

[16] Bamber A.I., Neal T.J. (1999). An Assessment of Triclosan Susceptibility in Methicillin-Resistant and Methicillin-Sensitive Staphylococcus Aureus. J. Hosp. Infect..

[17] Bovicelli P., Bernini R., Antonioletti R., Mincione E. (2002). Selective Halogenation of Flavanones. Tetrahedron Lett..

[18] Podgoršek A., Zupan M., Iskra J. (2009). Oxidative Halogenation with “Green” Oxidants: Oxygen and Hydrogen Peroxide. Angew. Chem. Int. Ed..

[19] Lu K., Chu J., Wang H., Fu X., Quan D., Ding H., Yao Q., Yu P. (2013). Regioselective Iodination of Flavonoids by N-Iodosuccinimide under Neutral Conditions. Tetrahedron Lett..

[20] Glenn W.S., Nims E., O’Connor S.E. (2011). Reengineering a Tryptophan Halogenase To Preferentially Chlorinate a Direct Alkaloid Precursor. J. Am. Chem. Soc..

[21] Frese M., Sewald N. (2015). Enzymatic Halogenation of Tryptophan on a Gram Scale. Angew. Chem. Int. Ed..

[22] Andorfer M.C., Belsare K.D., Girlich A.M., Lewis J.C. (2017). Aromatic Halogenation by Using Bifunctional Flavin Reductase–Halogenase Fusion Enzymes. Chembiochem.

[23] Sheldon R.A., van Pelt S. (2013). er Enzyme Immobilisation in Biocatalysis: Why, What and How. Chem. Soc. Rev..

[24] Sheldon R.A., Brady D. (2019). Broadening the Scope of Biocatalysis in Sustainable Organic Synthesis. ChemSusChem.

[25] Ludewig H., Molyneux S., Ferrinho S., Guo K., Lynch R., Gkotsi D.S., Goss R.J. (2020). Halogenases: Structures and Functions. Curr. Opin. Struc. Biol..

[26] Yeh E., Blasiak L.C., Koglin A., Drennan C.L., Walsh C.T. (2007). Chlorination by a Long-Lived Intermediate in the Mechanism of Flavin-Dependent Halogenases. Biochemistry.

[27] Pée K.H.V., Patallo E.P. (2006). Flavin-Dependent Halogenases Involved in Secondary Metabolism in Bacteria. Appl. Microbiol. Biot..

[28] Bitto E., Huang Y., Bingman C.A., Singh S., Thorson J.S., Phillips G.N. (2008). The Structure of Flavin-dependent Tryptophan 7-halogenase RebH. Proteins Struct. Funct. Bioinform..

[29] Menon B.R.K., Brandenburger E., Sharif H.H., Klemstein U., Shepherd S.A., Greaney M.F., Micklefield J. (2017). RadH: A Versatile Halogenase for Integration into Synthetic Pathways. Angew. Chem.-Ger. Ed..

[30] Quideau S., Deffieux D., Douat-Casassus C., Pouységu L. (2011). Pflanzliche Polyphenole: Chemische Eigenschaften, Biologische Aktivität Und Synthese. Angew. Chem.-Ger. Ed..

[31] Betts J.W., Sharili A.S., Phee L.M., Wareham D.W. (2015). In Vitro Activity of Epigallocatechin Gallate and Quercetin Alone and in Combination versus Clinical Isolates of Methicillin-Resistant Staphylococcus Aureus. J. Nat. Prod..

[32] Zizkova P., Stefek M., Rackova L., Prnova M., Horakova L. (2017). Novel Quercetin Derivatives: From Redox Properties to Promising Treatment of Oxidative Stress Related Diseases. Chem.-Biol. Interact..

[33] Gonzalez O., Fontanes V., Raychaudhuri S., Loo R., Loo J., Arumugaswami V., Sun R., Dasgupta A., French S.W. (2009). The Heat Shock Protein Inhibitor Quercetin Attenuates Hepatitis C Virus Production. Hepatology.

[34] Cushnie T.P.T., Lamb A.J. (2005). Antimicrobial Activity of Flavonoids. Int. J. Antimicrob. Agents.

[35] Ferrer J., Jez J.M., Bowman M.E., Dixon R.A., Noel J.P. (1999). Structure of Chalcone Synthase and the. Molecular Basis of Plant Polyketide Biosynthesis. Nat. Am. Inc..

[36] Myronovskyi M., Rosenkränzer B., Nadmid S., Pujic P., Normand P., Luzhetskyy A. (2018). Generation of a Cluster-Free Streptomyces Albus Chassis Strains for Improved Heterologous Expression of Secondary Metabolite Clusters. Metab. Eng..

[37] Buckingham J. (1993). Dictionary of Natural Products.

[38] Khattab A.I., Babiker E.H., Saeed H.A. (2016). Streptomyces: Isolation, Optimization of Culture Conditions and Extraction of Secondary Metabolites. Int. Curr. Pharm. J..

[39] Weber T., Blin K., Duddela S., Krug D., Kim H.U., Bruccoleri R., Lee S.Y., Fischbach M.A., Müller R., Wohlleben W. (2015). AntiSMASH 3.0—A Comprehensive Resource for the Genome Mining of Biosynthetic Gene Clusters. Nucleic Acids Res..

[40] Medema M.H., Blin K., Cimermancic P., de Jager V., Zakrzewski P., Fischbach M.A., Weber T., Takano E., Breitling R. (2011). AntiSMASH: Rapid Identification, Annotation and Analysis of Secondary Metabolite Biosynthesis Gene Clusters in Bacterial and Fungal Genome Sequences. Nucleic Acids Res..

[41] Siegl T., Tokovenko B., Myronovskyi M., Luzhetskyy A. (2013). Design, Construction and Characterisation of a Synthetic Promoter Library for Fine-Tuned Gene Expression in Actinomycetes. Metab. Eng..

[42] Ribeiro D., Freitas M., Lima J.L.F.C., Fernandes E. (2015). Proinflammatory Pathways: The Modulation by Flavonoids. Med. Res. Rev..

[43] Park H., Dao T.T., Kim H.P. (2005). Synthesis and Inhibition of PGE2 Production of 6,8-Disubstituted Chrysin Derivatives. Eur. J. Med. Chem..

[44] Marzec E., Świtalska M., Winiewska-Szajewska M., Wójcik J., Wietrzyk J., Maciejewska A.M., Poznański J., Mieczkowski A. (2020). The Halogenation of Natural Flavonoids, Baicalein and Chrysin, Enhances Their Affinity to Human Protein Kinase CK2. IUBMB Life.

[45] Zheng X., Meng W.-D., Xu Y.-Y., Cao J.-G., Qing F.-L. (2003). Synthesis and Anticancer Effect of Chrysin Derivatives. Bioorg. Med. Chem. Lett..

[46] Yaipakdee P., Robertson L.W. (2001). Enzymatic Halogenation of Flavanones and Flavones. Phytochemistry.

[47] Schnepel C., Minges H., Frese M., Sewald N. (2016). A High-Throughput Fluorescence Assay to Determine the Activity of Tryptophan Halogenases. Angew. Chem. Int. Ed..

[48] Yin L., Zhang Z., Wang Y. (2006). PEG (300)–PdCl2 Promoted Efficient and Convenient Suzuki–Miyaura Coupling of Aryl Chlorides with Arylboronic Acids. Tetrahedron.

[49] Unversucht S., Hollmann F., Schmid A., van Pée K. (2005). FADH2-Dependence of Tryptophan 7-Halogenase. Adv. Synth. Catal..

[50] Sanchez C., Butovich I.A., Brana A.F., Rohr J., Méndez C., Salas J.A. (2002). The Biosynthetic Gene Cluster for the Antitumor Rebeccamycin: Characterization and Generation of Indolocarbazole Derivatives. Chem. Biol..

[51] Latimer R., Podzelinska K., Soares A., Bhattacharya A., Vining L.C., Jia Z., Zechel D.L. (2009). Expression, Purification and Preliminary Diffraction Studies of CmlS. Acta Cryst. Sect. F Struct. Biol. Cryst. Commun..

[52] Payne J.T., Andorfer M.C., Lewis J.C. (2013). Regioselective Arene Halogenation Using the FAD-Dependent Halogenase RebH. Angew. Chem. Int. Ed..

[53] Yeh E., Cole L.J., Barr E.W., Bollinger J.M., Ballou D.P., Walsh C.T. (2006). Flavin Redox Chemistry Precedes Substrate Chlorination during the Reaction of the Flavin-Dependent Halogenase RebH. Biochemistry.

[54] Dong C., Flecks S., Unversucht S., Haupt C., Pée K.H.V., Naismith J.H. (2005). Structural Biology: Tryptophan 7-Halogenase (PrnA) Structure Suggests a Mechanism for Regioselective Chlorination. Science.

[55] Waterhouse A., Bertoni M., Bienert S., Studer G., Tauriello G., Gumienny R., Heer F.T., de Beer T.A.P., Rempfer C., Bordoli L. (2018). SWISS-MODEL: Homology Modelling of Protein Structures and Complexes. Nucleic Acids Res..

[56] Buedenbender S., Rachid S., Müller R., Schulz G.E. (2009). Structure and Action of the Myxobacterial Chondrochloren Halogenase CndH: A New Variant of FAD-Dependent Halogenases. J. Mol. Biol..

[57] Zhang Y., Skolnick J. (2005). TM-Align: A Protein Structure Alignment Algorithm Based on TM-Score. Nucleic Acids Res..

[58] (2021). Molecular Operating Enviroment (MOE), 2019.01.

[59] Green M.R., Sambrook J. (2012). Molecular Cloning: A Laboratory Manual.

[60] Rebets Y., Kormanec J., Luzhetskyy A., Bernaerts K., Anné J. (2016). Metagenomics, Methods and Protocols. Methods Mol. Biol..

[61] Flett F., Mersinias V., Smith C.P. (1997). Mersinias, V.; Smith, C.P. High Efficiency Intergeneric Conjugal Transfer of Plasmid DNA from *Escherichia coli* to Methyl DNA-restricting *Streptomycetes*. FEMS Microbiol. Lett..

[62] Kieser T., Buttner M.J., Carter K.F., Hopwood D.A. (2000). Practical Streptomyces Genetics.

[63] Kearse M., Moir R., Wilson A., Stones-Havas S., Cheung M., Sturrock S., Buxton S., Cooper A., Markowitz S., Duran C. (2012). Geneious Basic: An Integrated and Extendable Desktop Software Platform for the Organization and Analysis of Sequence Data. Bioinformatics.

[64] Altschul S.F., Wootton J.C., Gertz E.M., Agarwala R., Morgulis A., Schäffer A.A., Yu Y. (2005). Protein Database Searches Using Compositionally Adjusted Substitution Matrices. FEBS J..

[65] Notredame C., Higgins D.G., Heringa J. (2000). T-Coffee: A Novel Method for Fast and Accurate Multiple Sequence Alignment. J. Mol. Biol..

[66] Schrödinger L.L.C. (2015). The PyMOL Molecular Graphics System.

