# Supplementary material for: A Promiscuous Halogenase for the Derivatization of Flavonoids
Source: Molecules. 2021 Oct 14;26(20):6220. doi: 10.3390/molecules26206220 (PMC8539768; doi:10.3390/molecules26206220)
Supplement: Supplementary file 1 [file molecules-26-06220-s001.zip › molecules-1395360-supplementary.pdf]

molecules

Supplementary Information

Promiscuous Halogenase for Derivatization of Flavonoids

Dominik Kolling [1], Marc Stierhof [1], Constanze Lasch [1], Maksym Myronovskyi [1] and Andriy Luzhetskyy [1,2,*]

[1] Pharmaceutical Biotechnology, Saarland University, 66123 Saarbruecken, Germany; kolling-dominik@gmail.com (D.K.); m.stierhof@t-online.de (M.S.); constanze.lasch@uni-saarland.de (C.L.); maksym.myronovskyi@uni-saarland.de (M.M.)
[2] Helmholtz Institute for Pharmaceutical Research Saarland, 66123 Saarbruecken, Germany
* Correspondence: a.luzhetskyy@mx.uni-saarland.de; Tel.: +49-681-302-70200

Content

Page S2: Supplementary Tables
Page S3-7: Supplementary Figures
Page S8-13: HPLC-MS data
Page S14-20: NMR data

Supplementary Tables

Table S1. Bacterial strains, fosmids, plasmids and primers used in this work.

**Bacterial strain**	**Features**	**Reference/Source**
Streptomyces albus Del14	Wild-type strain	[1]
Streptomyces albus 9B-A9	*S. albus* with BAC 9B-A9 insertion	This work
Streptomyces albus pSETprom-Hal	*S. albus* with pSETprom-Hal insertion	This work
Escherichia coli ET12567 pUB307	Donor strain for intergeneric conjugation	[2]
Escherichia coli GB2005	General cloning strain	-
**fosmid/plasmid**	**Features**	**Reference/Source**
9B-A9	BAC containing DklH gene	[1]
pSET152	AmR, cloning vector	-
**Primers**	**Sequence (5´-3´)**	**Source**
pSETprom-Hal_F	AAAATCTAGA<u>CAGGTCGGCTGGTT-GGCTGACAAAGTGCGTAGGATGACTGT</u>CCTCTCG-GACAGGCTGTCGA	Eurofines Scientific
pSETprom-Hal_R	AAAAGATATCCTACTGGAGGTTGGCGAGCC	Eurofines Scientific
halseqF1	CTGGAAAAATGCGACGCCG	Eurofines Scientific
halseqF2	CAGCAGAACCAGGGGAAGG	Eurofines Scientific
halseqR1	GACAACAGCGGGTCCAGAA	Eurofines Scientific
halseqR2	TCTCGGCGTTGGTGATTCC	Eurofines Scientific

Supplementary Figures

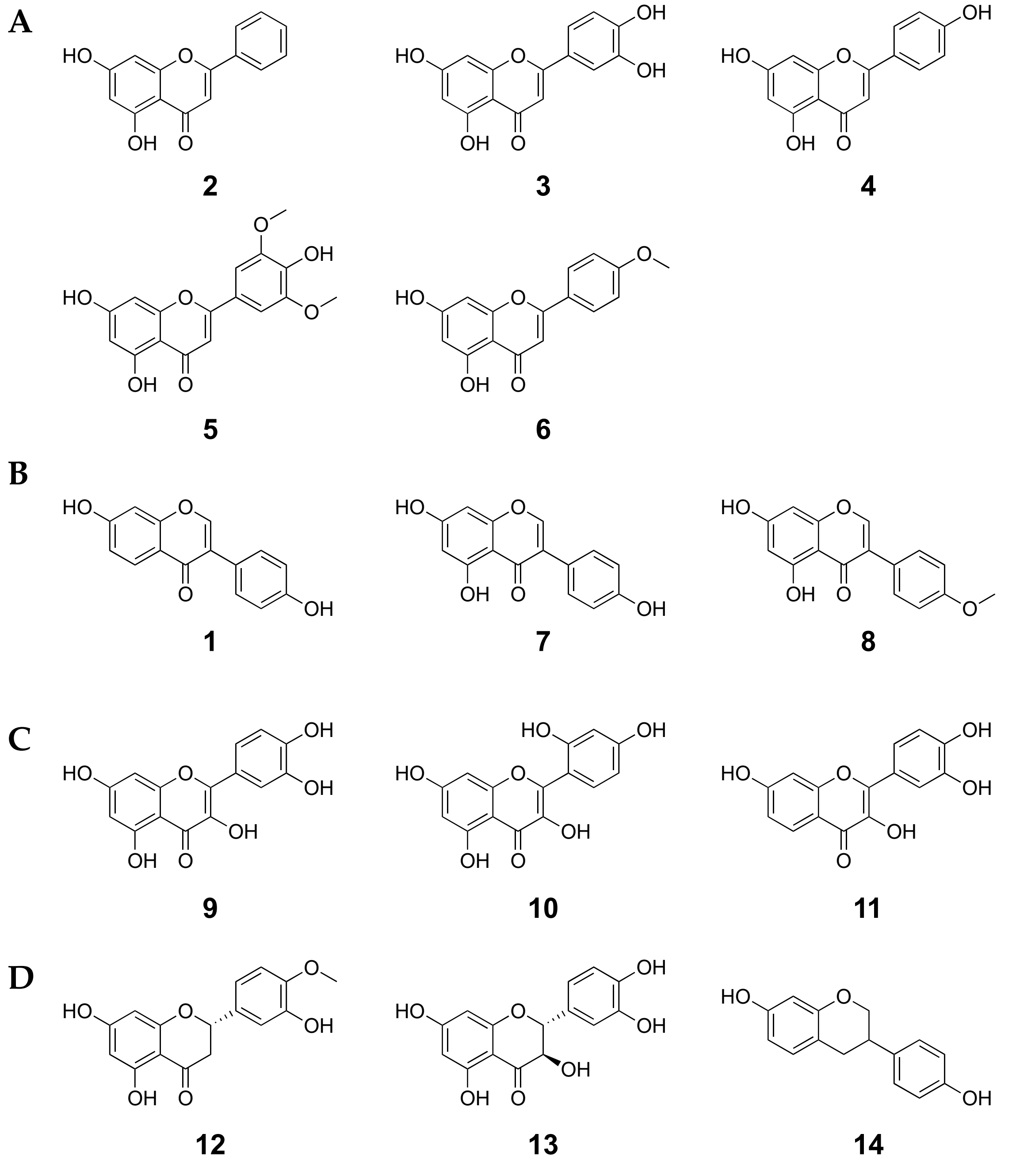

Figure S1. Outline of DklH substrates classified by flavonoid substructure. (**A**) Flavone: chrysin[d] **2**, luteolin[b] **3**, apigenin[b] **4**, tricin[c] **5**, acacetin[c] **6**. (**B**) Isoflavone: daidzein[b] **1**, genistein[b] **7**, biochanin A[a] **8**. (**C**) Flavanonole: quercetin[b] **9**, morin[b] **10**, fisetin[a] **11**. (**D**) Flavanone: hesperetin[b] **12**, flavanonole: taxifolin[b] **13** and isoflavandiol: R,S-equol[b] **14**. Suppliers: (a) TCI Germany GmbH, Eschborn, Germany; (b) Santa Cruz Biotechnology, Inc., Dallas, TX, USA; (c) SynInnova Laboratories Inc., Edmonton, Canada; (d) Alfa Aesar, Thermo Fisher Scientific, Kandel, Germany.

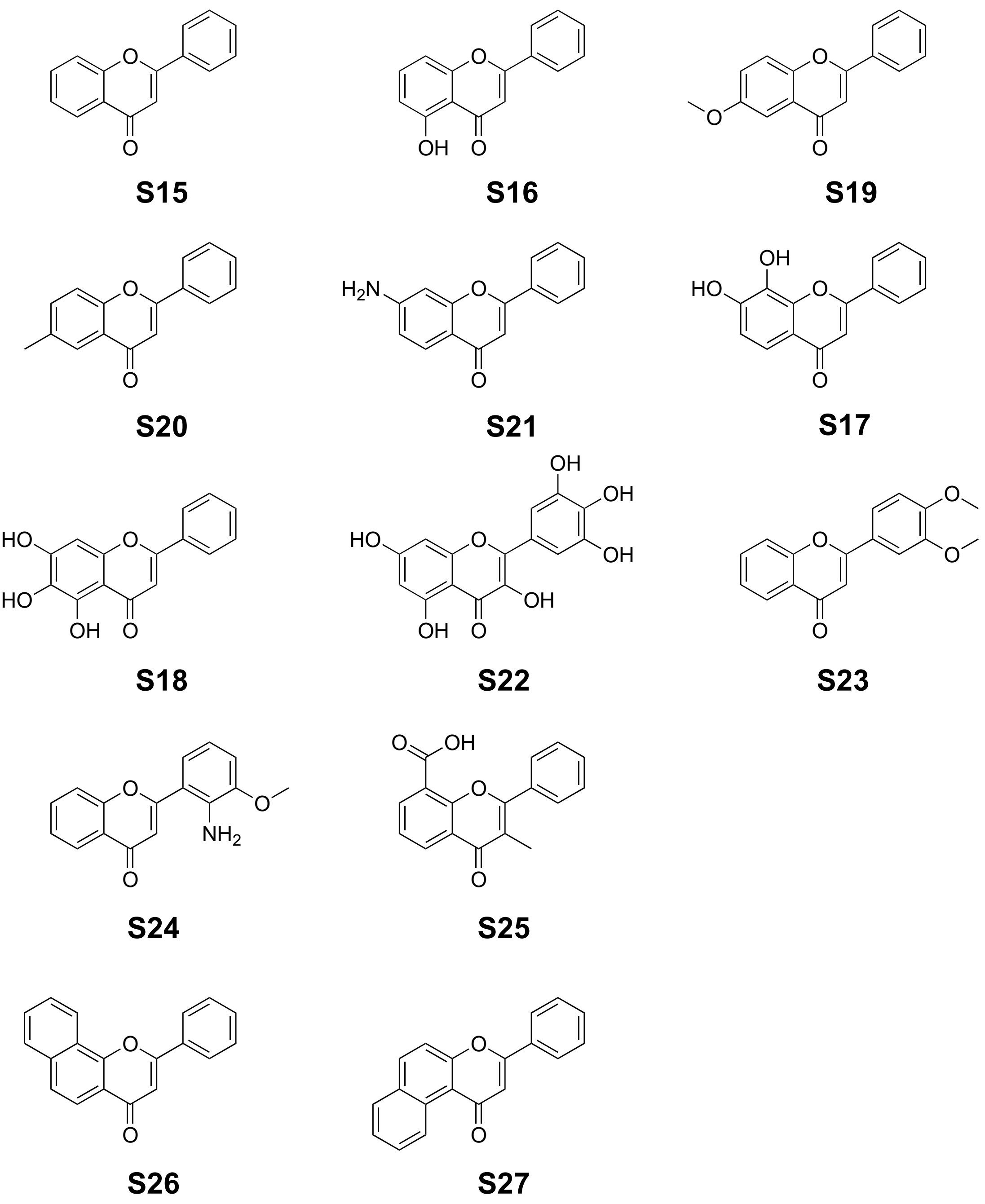

Figure S2. Display of flavones and related derivatives not accepted by DklH. Flavone[a] **S15**, 6-hydroxyflavone[a] **S16**, 6-methoxyflavone[a] (**S19**), 6-methylflavone[a] **S20**, 7-aminoflavone[b] **S21**, 7,8-dihydroxyflavone[b] **S17**, baicalein[b] **S18**, myricetin[a] **S22**, 3′,4′-dimethoxyflavone[a] **S23**, 2-(2-amino-3-methoxyphenyl)chromone[a] **S24**, 3-methylflavone-8-carboxylic acid[a] **S25**, α-naphthoflavone[a] **S26**, β-naphthoflavone[a] **S27**. Suppliers: (a) TCI Germany GmbH, Eschborn, Germany; (b) Santa Chruz Biotechnology, Inc., Dallas, TX, USA.

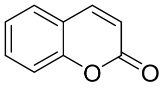

S28

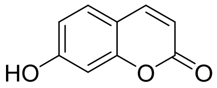

S29

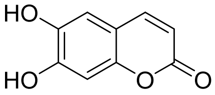

S30

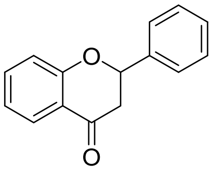

S31

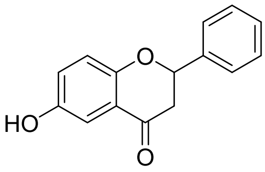

S32

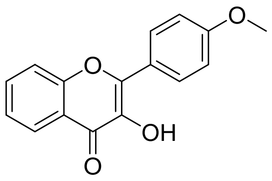

S33

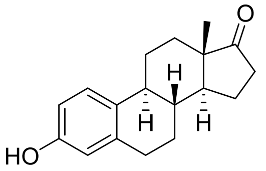

S34

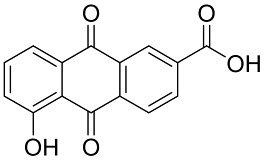

S35

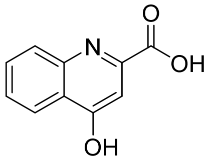

S36

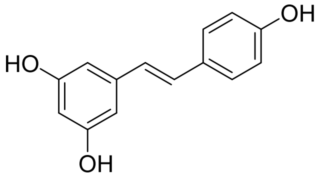

S37

Figure S3. Display of compounds not converted by DklH. Coumarin[a] **S28**, umbelliferone[a] **S29**, esculetin[a] **S30**, flavanone[a] **S31**, 6-hydroxyflavanone[a] **S32**, 3-hydroxy-4′-methoxyflavone[a] **S33**, estron[a] **S34**, 4,5-dihydroxyanthraquinone-2-carboxylic acid[a] **S35**, kynurenic acid[a] **S36**, resveratrol[a] **S37**. Suppliers: (a) TCI Germany GmbH, Eschborn, Germnay; (b) Santa Chruz Biotechnology, Inc., Dallas, TX, USA.

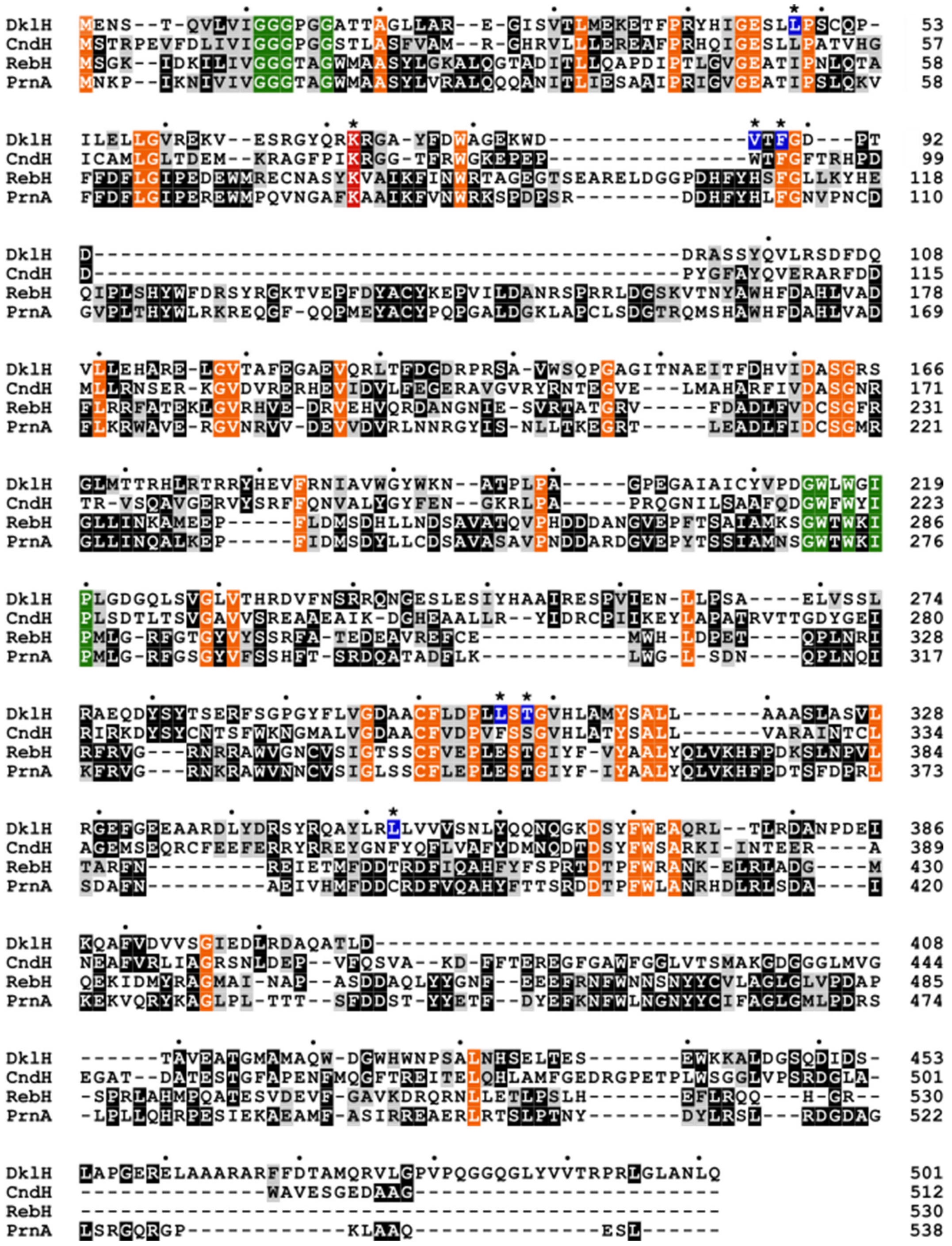

Figure S4. Multiple sequence alignment between DklH from *Frankia alni* ACN14a, chondrochloren halogenase. (CndH) from *Chondromyces crocatus* Cm c5, flavin-dependent tryptophan halogenase (RebH) from *Lechevalieria aerocolonigenes* and flavin-dependent tryptophan halogenase (PrnA) from *Pseudomonas fluorescens*. The sequence alignment was performed using the T-Coffee server [3]. The results were visualized by using Boxshade version 3.21. Every tenth residue of DklH is indicated by a dot above. Conserved regions are highlighted by different colors. Overall conserved residues are represented in orange. The FAD binding site is shown in green, while the motif GGGXXG indicates diphosphate binding residues. The FAD isoalloxazine-ring is coordinated by GWXWXIP substructure [4]. Residues that are involved in substrate binding are shown in blue and are additionally labelled with a star above. The lysin-72, which transfers activated chlorine species to substrate, is colored in red.

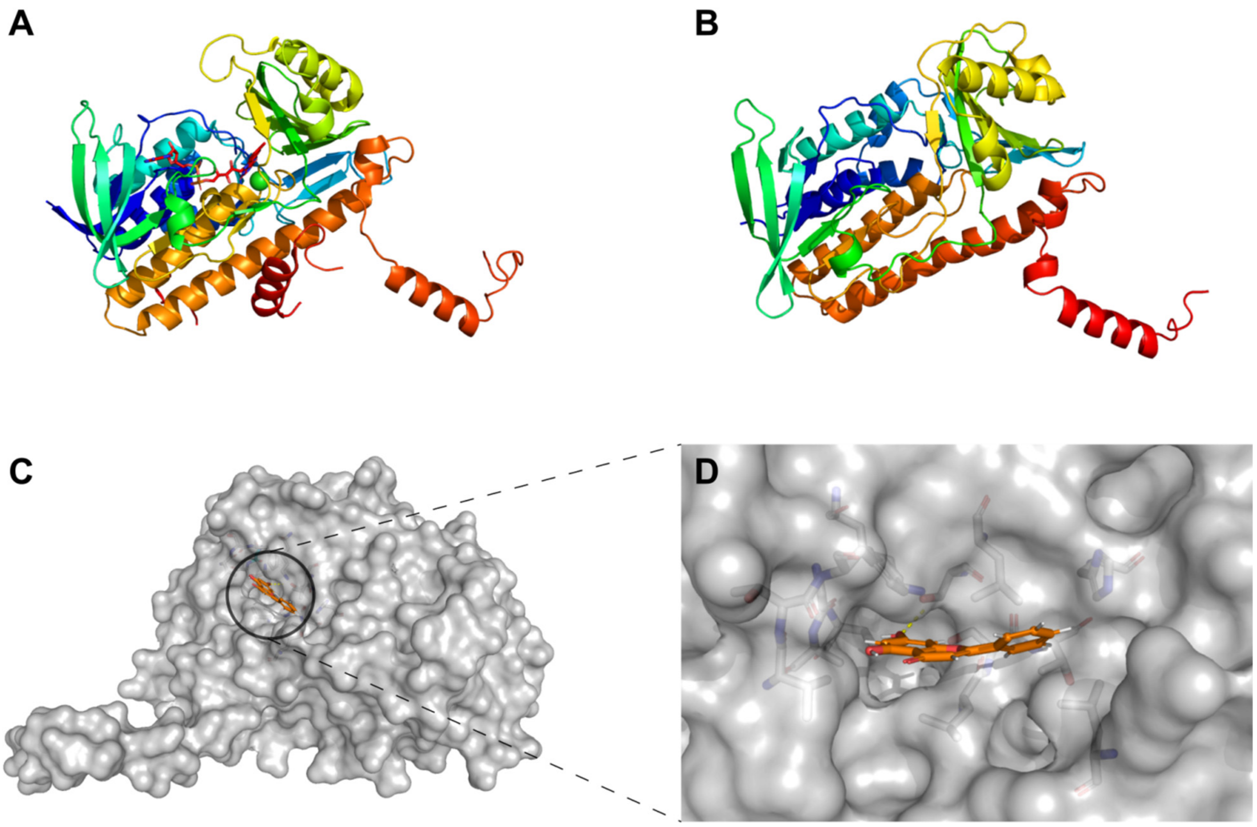

Figure S5. (**A**) Crystal structure of chondrochloren halogenase (CndH) from *Chondromyces crocatus* Cm c5 (PDB code 3E1T) in complex with the encased FAD (red) represented as sticks. Chloride (green) is shown as dot near to FAD binding site and active lysin-72 residue. (**B**) Derived homology model of DklH based on the crystal structure of CndH. (**C**) Surface representation of DklH homology model with the bound substrate chrysin. (**D**) Detailed view on substrate binding site of DklH. The ligand **2** (orange) is situated in a non-polar cavity of the protein. The A ring of flavonoid has deeply access to the narrow region near to active lysin-72, which transfers the chlorine to substrate. Therefor the putative binding mode correlates with observed halogenation pattern.

HPLC-MS data

The LC-MS chromatograms of crude extracts are displayed for the feeding experiments of *S. albus* Del14 9B-A9 cultures supplemented with flavonoid in concentration of 0.02 mg/L in DM media. In the parallel experiment the feeding was also carried out with the controle strains *S. albus* Del14 and *S. albus* Del14 pSET-DklH under the same conditions (data not shown). The chromatograms shown below are represented as extractet ion chromatograms only highlighting the peaks with the corresponding mass of interest. The chlorinated deriviatives were identified by exact mass and isotope peaks. The exact position of halogen was nearly estimated by determination of the relative retention time (Δt_R) compared to unmodified flavonoid. Therefore the NMR data and chromatograms for chrysin **2** feeding revealed that a relative retention time of Δt_R = 0.7 min correlates with halogenation in position 6 (**2a**). Otherwise the relative retention time of Δt_R = 1.2 min indicates chlorination in position 8 (**2b**).

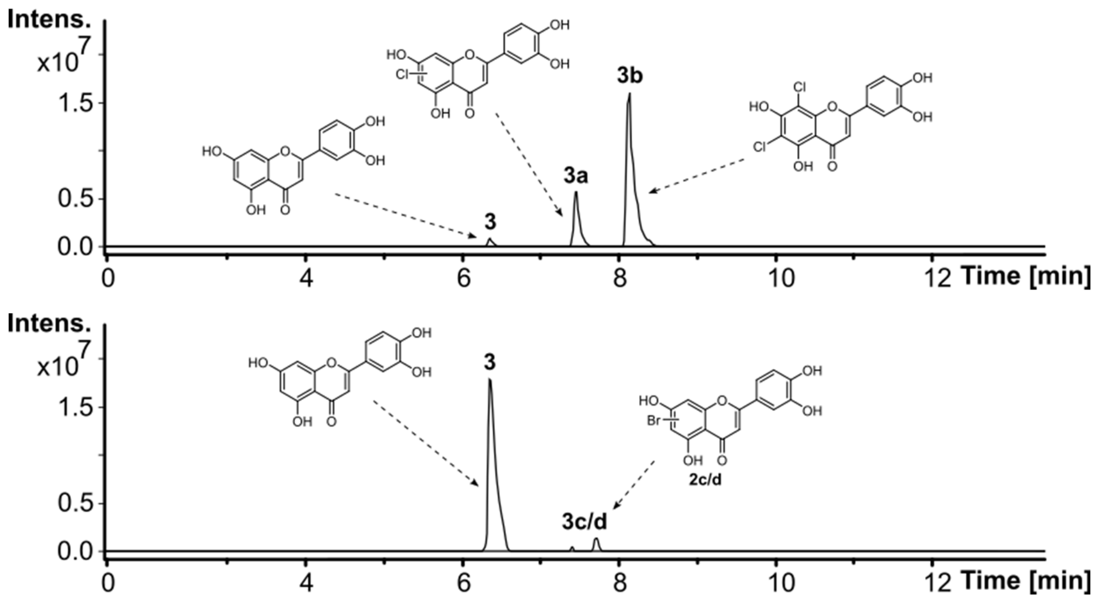

Figure S6. Extracted ion chromatogram of luteolin feeding experiment. The unmodified luteolin **3** (t_R = 6.4 min, $[M+H]^+_{cal.}$ = 287.0550 m/z, $[M+H]^+_{obs.}$ = 287.0551 m/z) occurs only in small amounts. Due to relative retention time Δt_R = 1.1 min the single chlorinated derivative **3a** (t_R = 7.5 min, $[M+H]^+_{cal.}$ = 321.0160 m/z, $[M+H]^+_{obs.}$ = 321.0160 m/z) may correspond with 8-Cl-luteolin. The doubly chlorinated compound **3b** (t_R = 8.2 min, $[M+H]^+_{cal.}$ = 354.9771 m/z, $[M+H]^+_{obs.}$ = 354.9722 m/z) strongly indicates halogenation in positions 6 and 8. The brominated compounds **3c** (t_R = 7.2 min, $[M+H]^+_{cal.}$ = 364.9655 m/z, $[M+H]^+_{obs.}$ = 364.9661 m/z) and **3d** (t_R = 7.7 min, $[M+H]^+_{cal.}$ = 364.9655 m/z, $[M+H]^+_{obs.}$ = 364.9661 m/z) are shown below.

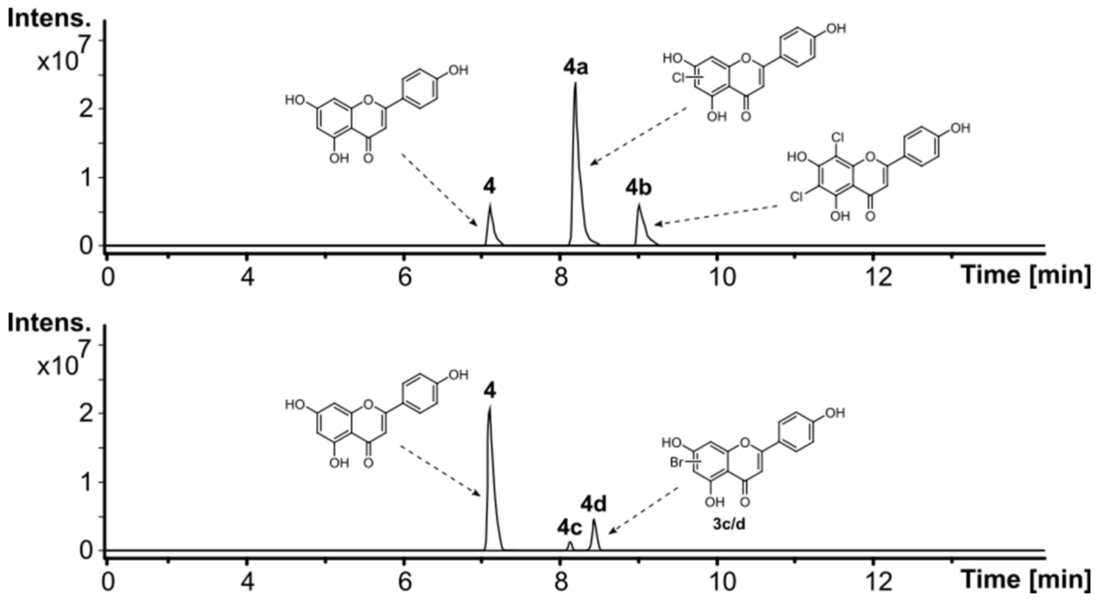

Figure S7. Extracted ion chromatogram of apigenin feeding experiment. Compound **4** (t_R = 7.1 min, $[M+H]^+_{cal.}$ = 271.0601 m/z, $[M+H]^+_{obs.}$ = 271.0599 m/z) is equivalent to apigenin. The relative retention time Δt_R = 1.1 min of compound **4a** (t_R = 8.2 min, $[M+H]^+_{cal.}$ = 305.0211 m/z, $[M+H]^+_{obs.}$ = 305.0213 m/z) indicates chlorination in position 8. The doubly chlorinated compound **4b** (t_R = 9.0 min, $[M+H]^+_{cal.}$ = 338.9822 m/z, $[M+H]^+_{obs.}$ = 338.9823 m/z) was proposed as 6,8-di-Cl-apigenin. The brominated derivatives of apigenin **4c** (t_R = 8.2 min, $[M+H]^+_{cal.}$ = 348.9706 m/z, $[M+H]^+_{obs.}$ = 348.9712 m/z) and **4d** (t_R = 8.5 min, $[M+H]^+_{cal.}$ = 348.9706 m/z, $[M+H]^+_{obs.}$ = 348.9711 m/z) are shown below.

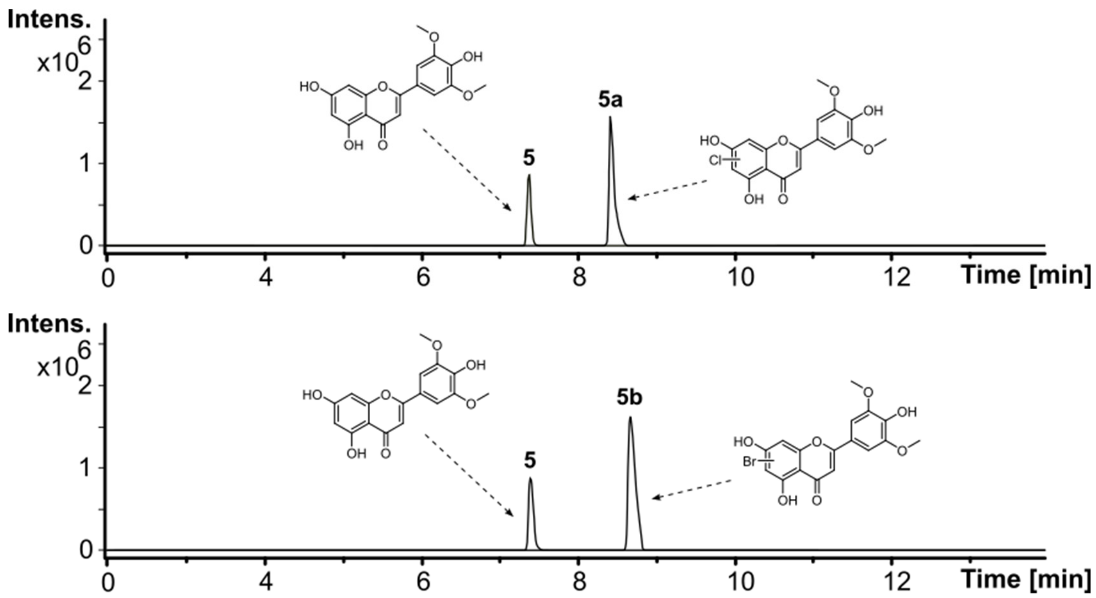

Figure S8. Extracted ion chromatogram of tricin feeding experiment. The O-methylated compound **5** (t_R = 7.4 min, $[M+H]^+_{cal.}$ = 331.0812 m/z, $[M+H]^+_{obs.}$ = 331.0818 m/z) was identified as tricin. As chlorination product, only compound **5a** (t_R = 8.4 min, $[M+H]^+_{cal.}$ = 365.0423 m/z, $[M+H]^+_{obs.}$ = 365.0419 m/z) with one chlorine at suggested position 8 (Δt_R = 1.0 min) was found. The double chlorinated derivative of tricin is missing. The brominated analog **5b** (t_R = 8.7 min, $[M+H]^+_{cal.}$ = 408.9917 m/z, $[M+H]^+_{obs.}$ = 408.9919 m/z) was obtained in reasonable yield.

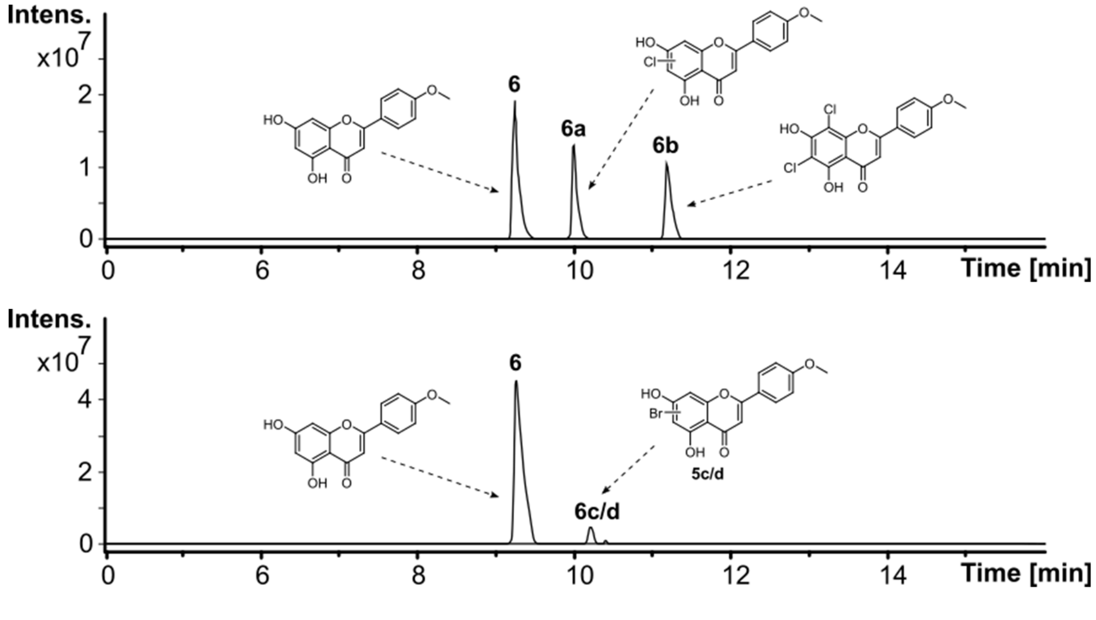

Figure S9. Extracted ion chromatogram of acacetin feeding experiment. The peak with strongest intensity corresponds with acacetin **6** (t_R = 9.3 min, $[M+H]^+_{cal.}$ = 285.0757 m/z, $[M+H]^+_{obs.}$ = 285.0763 m/z). Due to calculation of relative retention time Δt_R = 0.7 min, the compound **6a** (t_R = 10.0 min, $[M+H]^+_{cal.}$ = 319.0368 m/z, $[M+H]^+_{obs.}$ = 319.0369 m/z) was proposed as 6-Cl-acacetin. The derivative **6b** (t_R = 11.2 min, $[M+H]^+_{cal.}$ = 352.9978 m/z, $[M+H]^+_{obs.}$ = 352.9982 m/z) was therefore identified as doubly chlorinated reaction product 6,8-di-Cl-acacetin. The brominated acacetin analogs **6c** (t_R = 10.2 min, $[M+H]^+_{cal.}$ = 362.9863 m/z, $[M+H]^+_{obs.}$ = 362.9863 m/z) and **6d** (t_R = 10.6 min, $[M+H]^+_{cal.}$ = 362.9863 m/z, $[M+H]^+_{obs.}$ = 362.9860 m/z) are present in very small quantities.

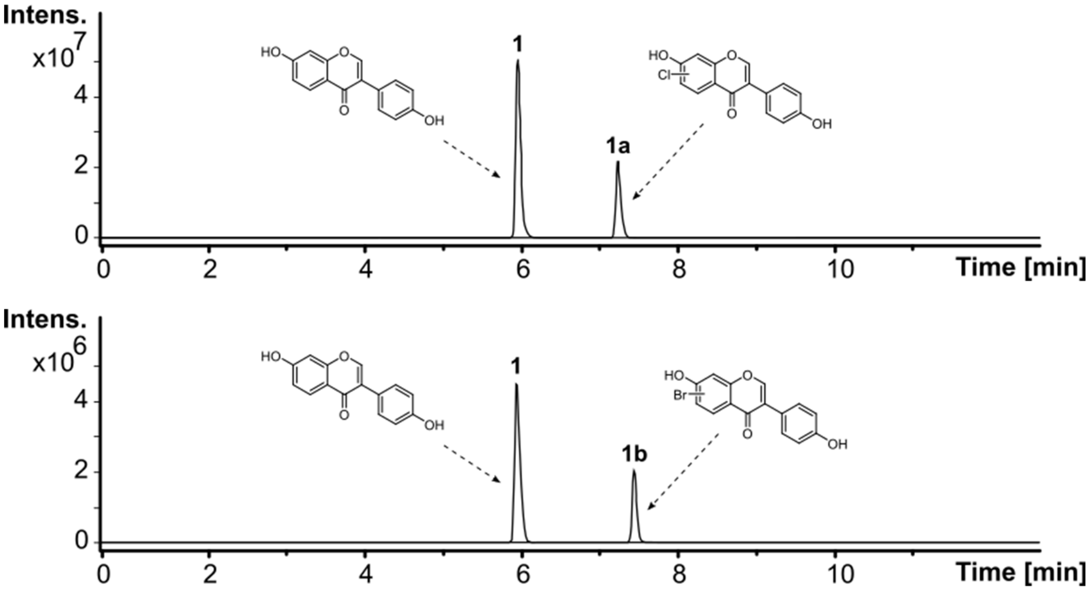

Figure S10. Extracted ion chromatogram of daidzein feeding experiment. Compound **1** (t_R = 6.0 min, $[M+H]^+_{cal.}$ = 255.0652 m/z, $[M+H]^+_{obs.}$ = 255.0655 m/z) was identified as daidzein. The structure of chlorine containing derivative **1a** (t_R = 7.2 min, $[M+H]^+_{cal.}$ = 289.0262 m/z, $[M+H]^+_{obs.}$ = 289.0266 m/z) was suggested as 8-Cl-daidzein (Δt_R = 1.2 min) and confirmed by NMR analysis. The brominated analog **1b** (t_R = 7.5 min, $[M+H]^+_{cal.}$ = 332.9757 m/z, $[M+H]^+_{obs.}$ = 332.9760 m/z) is represented below.

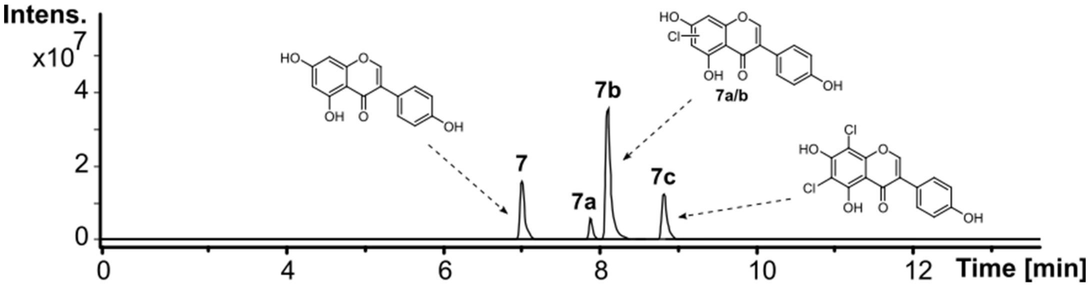

Figure S11. Extracted ion chromatogram of genistein feeding experiment. The unmodified compound **7** (t_R = 7.0 min, $[M+H]^+_{cal.}$ = 271.0601 m/z, $[M+H]^+_{obs.}$ = 271.0602 m/z) correlates with genistein. For halogenation two derivatives containing one chlorine were obtained. The calculation of relative retention time Δt_R = 0.9 min led to proposal that compound **7a** (t_R = 7.9 min, $[M+H]^+_{cal.}$ = 305.0211 m/z, $[M+H]^+_{obs.}$ = 305.0212 m/z) shows chlorination in position 6. In contrast to that the relative retention time for **7b** (t_R = 8.1 min, $[M+H]^+_{cal.}$ = 305.0211 m/z, $[M+H]^+_{obs.}$ = 305.0213 m/z) revealed 8-Cl-genistein as suggested product. The isotope peaks for derivative **7c** (t_R = 8.8 min, $[M+H]^+_{cal.}$ = 338.9822 m/z, $[M+H]^+_{obs.}$ = 338.9824 m/z) indicates double chlorination.

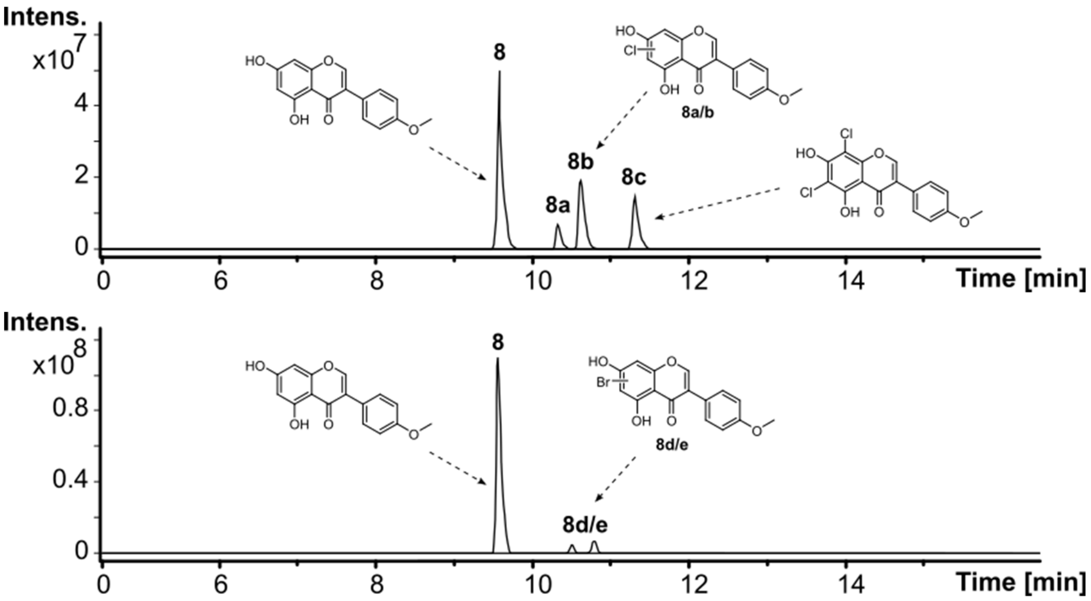

Figure S12. Extracted ion chromatogram of biochanin A feeding experiment. The peak with strongest intensity was identified as biochanin A **8** (t_R = 9.6 min, $[M+H]^+_{cal.}$ = 285.0757 m/z, $[M+H]^+_{obs.}$ = 285.0770 m/z). Due to relative retention time of Δt_R = 0.7 min the compound **8a** (t_R = 10.3 min, $[M+H]^+_{cal.}$ = 319.0368 m/z, $[M+H]^+_{obs.}$ = 319.0372 m/z) strongly corresponds with one chlorine containing derivative and halogenation in position 6. Compared to that compound **8b** (t_R = 10.6 min, $[M+H]^+_{cal.}$ = 319.0368 m/z, $[M+H]^+_{obs.}$ = 319.0376 m/z) was found in larger amount. The relative retention time of Δt_R = 1.0 min for this peak correlates with proposed 8-Cl-biochanin A. The derivative **8c** (t_R = 11.3 min, $[M+H]^+_{cal.}$ = 352.9978 m/z, $[M+H]^+_{obs.}$ = 352.9979 m/z) showed double incorporation of chlorine in the suggested position 6 and 8. The compounds **8d** (t_R = 10.5 min, $[M+H]^+_{cal.}$ = 362.9863 m/z, $[M+H]^+_{obs.}$ = 362.9867 m/z) and **8e** (t_R = 10.8 min, $[M+H]^+_{cal.}$ = 362.9863 m/z, $[M+H]^+_{obs.}$ = 362.9865 m/z) were identified as brominated analogs according to isotope peaks.

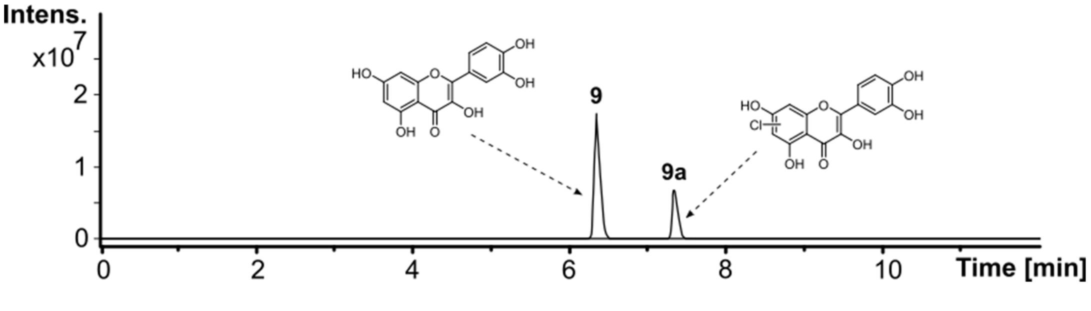

Figure S13. Extracted ion chromatogram of quercetin feeding experiment. The unmodified compound **9** (t_R = 6.3 min, $[M+H]^+_{cal.}$ = 303.0499 m/z, $[M+H]^+_{obs.}$ = 303.0500 m/z) was identified as quercetin. Chlorine containing derivative **9a** (t_R = 7.3 min, $[M+H]^+_{cal.}$ = 337.0110 m/z, $[M+H]^+_{obs.}$ = 337.0107 m/z) presumably corresponds with 8-Cl-quercetin (Δt_R = 1.0 min).

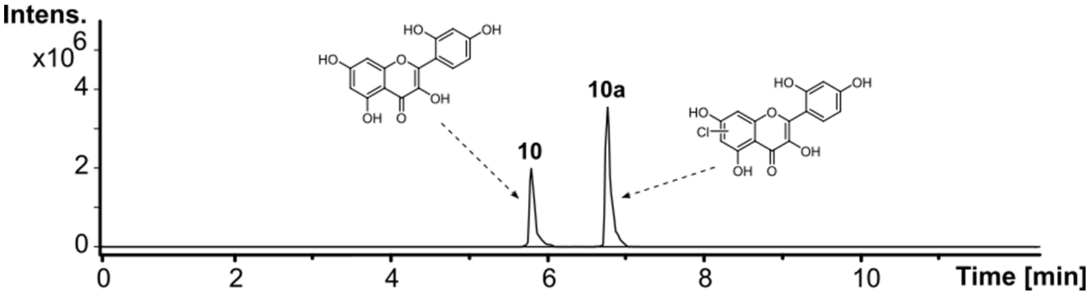

Figure S14. Extracted ion chromatogram of morin feeding experiment. Compound **10** (t_R = 5.8 min, $[M+H]^+_{cal.}$ = 303.0499 m/z, $[M+H]^+_{obs.}$ = 303.0501 m/z) was identified as morin. The related derivative **10a** (t_R = 6.8 min, $[M+H]^+_{cal.}$ = 337.0110 m/z, $[M+H]^+_{obs.}$ = 337.0108 m/z) with relative retention time of Δt_R = 1.0 min correlates with 8-Cl-morin.

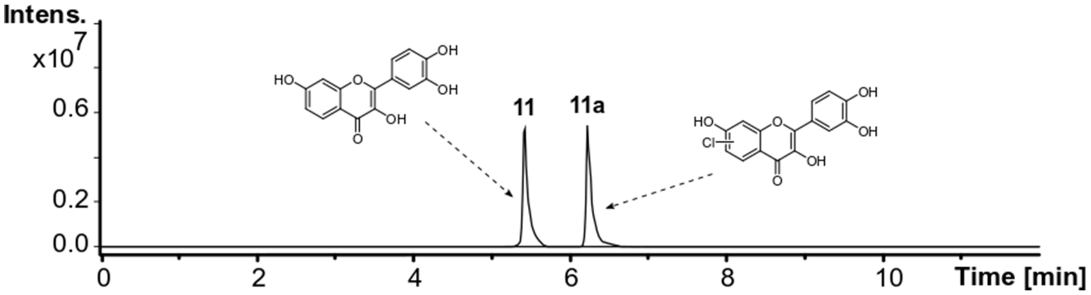

Figure S15. Extracted ion chromatogram of fisetin feeding experiment. For fisetin **11** (t_R = 5.4 min, $[M+H]^+_{cal.}$ = 287.0550 m/z, $[M+H]^+_{obs.}$ = 287.0550 m/z) the chlorine containing derivative **11a** (t_R = 6.2 min, $[M+H]^+_{cal.}$ = 321.0160 m/z, $[M+H]^+_{obs.}$ = 321.0162 m/z) was obtained in equal amounts. The proposed halogenation position 8 was identified due to relative retention time of Δt_R = 1.2 min.

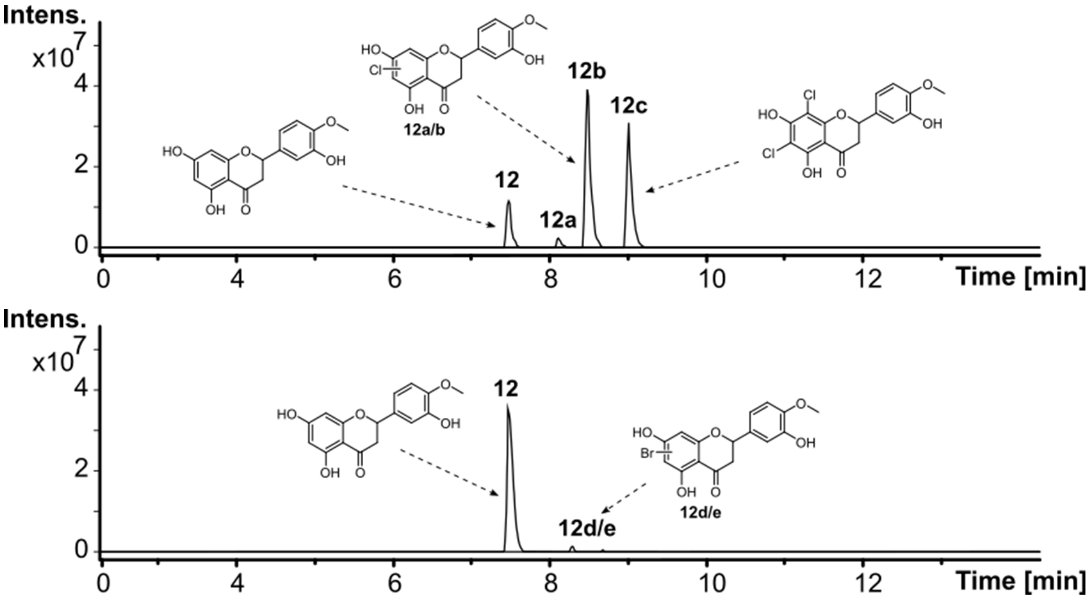

Figure S16. Extracted ion chromatogram of hesperetin feeding experiment. The first peak with small intensity **12** (t_R = 7.5 min, $[M+H]^+_{cal.}$ = 303.0863 m/z, $[M+H]^+_{obs.}$ = 303.0866 m/z) was identified as hesperetin. For halogenation two derivatives containing one chlorine in different positions were obtained. With aide of relative retention time of Δt_R = 0.6 min compound **12a** (t_R = 8.1 min, $[M+H]^+_{cal.}$ = 337.0473 m/z, $[M+H]^+_{obs.}$ = 337.0475 m/z) corresponds with proposed 6-Cl-hesperetin. Moreover derivative **12b** (t_R = 8.5 min, $[M+H]^+_{cal.}$ = 337.0473 m/z, $[M+H]^+_{obs.}$ = 337.0479 m/z) shows a relative retention time of Δt_R = 1.0 min which indicates chlorination in position 8. For double incorporation of chlorine compound **12c** (t_R = 9.0 min, $[M+H]^+_{cal.}$ = 371.0084 m/z, $[M+H]^+_{obs.}$ = 371.0088 m/z) was identified. The bromine containing derivatives **12d** (t_R = 8.3 min, $[M+H]^+_{cal.}$ = 380.9968 m/z, $[M+H]^+_{obs.}$ = 380.9966 m/z) and **12e** (t_R = 8.7 min, $[M+H]^+_{cal.}$ = 380.9968 m/z, $[M+H]^+_{obs.}$ = 380.9969 m/z) were detected in very small amounts.

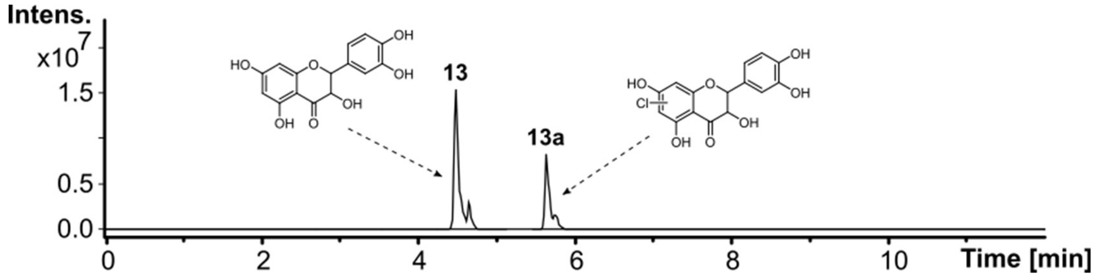

Figure S17. Extracted ion chromatogram of taxifolin feeding experiment. The first peak indicates presence of unmodified taxifolin **13** (t_R = 4.5 min, $[M+H]^+_{cal.}$ = 305.0656 m/z, $[M+H]^+_{obs.}$ = 305.0655 m/z). Compound **13a** (t_R = 5.7 min, $[M+H]^+_{cal.}$ = 339.0266 m/z, $[M+H]^+_{obs.}$ = 339.0267 m/z) was identified as 8-Cl-taxifolin according to relative retention time of Δt_R = 1.2 min.

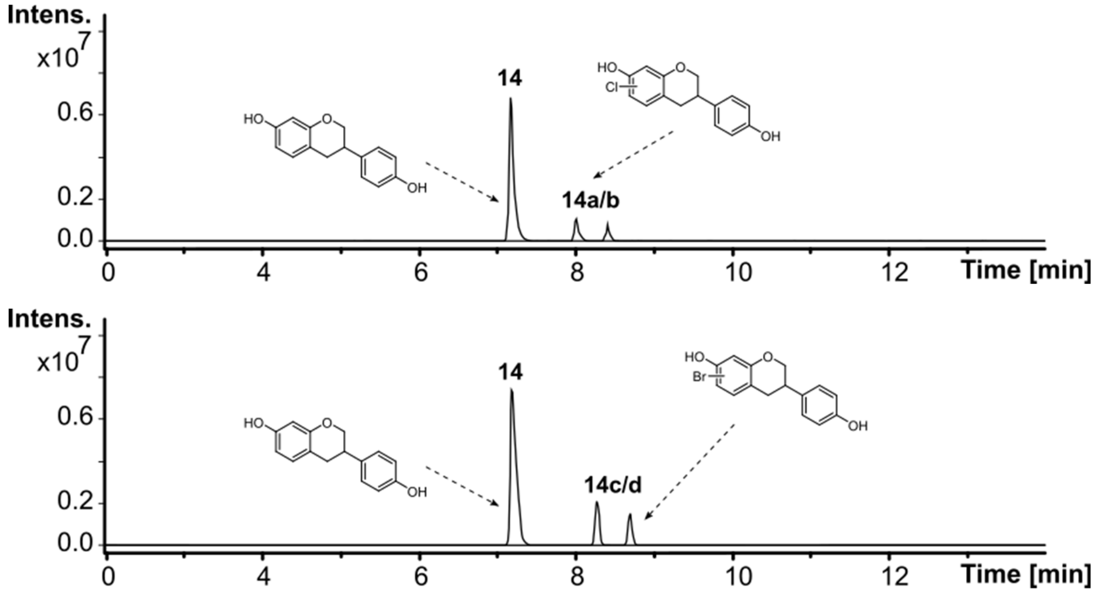

Figure S18. Extracted ion chromatogram of *R,S*-equol feeding experiment. The peak with strongest intensity **14** (t_R = 7.2 min, $[M+H]^+_{cal.}$ = 243.1016 m/z, $[M+H]^+_{obs.}$ = 243.1016 m/z) was identified as *R,S*-equol. Two reactions products were obtained for chlorination experiment as well as for bromination. Due to relative retention time of Δt_R = 0.8 min the compound **14a** (t_R = 8.0 min, $[M+H]^+_{cal.}$ = 277.0626 m/z, $[M+H]^+_{obs.}$ = 277.0624 m/z) correlates with proposed R,S-6-Cl-equol. The derivative **14b** (t_R = 8.4 min, $[M+H]^+_{cal.}$ = 277.0624 m/z, $[M+H]^+_{obs.}$ = 277.0624 m/z) with relative retention time of Δt_R = 1.2 min shows proposed chlorination in position 8. The compounds **14c** (t_R = 8.3 min, $[M+H]^+_{cal.}$ = 321.0121 m/z, $[M+H]^+_{obs.}$ = 321.0115 m/z) and **14d** (t_R = 8.7 min, $[M+H]^+_{cal.}$ = 321.0121 m/z, $[M+H]^+_{obs.}$ = 321.0120 m/z) represents the bromine containing flavonoids.

Table S2. LC-MS quantification and AUC determination for the chlorinated flavonoids. The data observed from the feeding experiment for the strain *S. albus* Del14 9B-A9 were calculated form UV absorption at 280 nm.

Flavonoid	6-Cl_1 [AUC]	8-Cl_1 [AUC]	6,8-Cl_2 [AUC]	Unmodified [AUC]
Chrysin (**2**)	40043	2669	6874	16617
Luteolin (**3**)	201684	n.d.	1420409	18617
Apigenin (**4**)	136715	n.d.	29021	12521
Tricin (**5**)	12525	n.d.	n.d.	7521
Acacetin (**6**)	27410	n.d.	37091	26545
Daidzein (**1**)	93633	n.d.	n.d.	284223
Genistein (**7**)	12100	107345	58138	28119
Biochanin A (**8**)	16755	63109	73077	129896
Quercetin (**9**)	19172	n.d.	n.d.	35531
Morin (**10**)	111159	n.d.	n.d.	42814
Fisetin (**11**)	10371	n.d.	n.d.	11142
Hesperetin (**12**)	23933	1216164	1127255	238997
Taxifolin (**13**)	15699	n.d.	n.d.	86157
R,S-Equol (**14**)	166125	56245	n.d.	1322615

Table S3. Yield [%] determination for the chlorinated flavonoids based on the estimated AUC

Flavonoid	6-Cl_1 [%]	8-Cl_1 [%]	6,8-Cl_2 [%]	Unmodified [%]
Chrysin (**2**)	60.8	4.0	10.3	24.9
Luteolin (**3**)	12.2	n.d.	86.6	1.1
Apigenin (**4**)	76.7	n.d.	16.3	7.0
Tricin (**5**)	62.5	n.d.	n.d.	37.5
Acacetin (**6**)	30.1	n.d.	40.7	29.2
Daidzein (**1**)	24.8	n.d.	n.d.	75.2
Genistein (**7**)	5.9	52.2	28.3	13.7
Biochanin A (**8**)	5.9	22.3	25.8	45.9
Quercetin (**9**)	35.0	n.d.	n.d.	65.0
Morin (**10**)	72.2	n.d.	n.d.	27.8
Fisetin (**11**)	48.2	n.d.	n.d.	51.8
Hesperetin (**12**)	9.2	46.7	43.3	9.2
Taxifolin (**13**)	15.4	n.d.	n.d.	84.6
R,S-Equol (**14**)	10.7	3.6	n.d.	85.6

Table S4. LC-MS quantification and AUC determination for the brominated flavonoids. The data observed from the feeding experiment for the strain *S. albus* Del14 9B-A9 were calculated form UV absorption at 280 nm.

Flavonoid	6-Br1 [AUC]	8-Br1 [AUC]	Unmodified [AUC]
Chrysin (**2**)	391945	53510	1000756
Luteolin (**3**)	894661	6332011	78843016
Apigenin (**4**)	24609	206991	750257
Tricin (**5**)	28030	n.d.	7521
Acacetin (**6**)	907588	0.09	8277759
Daidzein (**1**)	81874	n.d.	124570
Genistein (**7**)	Not tested	Not tested	Not tested
Biochanin A (**8**)	10723	14782	265075
Quercetin (**9**)	Not tested	Not tested	Not tested
Morin (**10**)	Not tested	Not tested	Not tested
Fisetin (**11**)	Not tested	Not tested	Not tested
Hesperetin (**12**)	26609	15665	1054121
Taxifolin (**13**)	Not tested	Not tested	Not tested
R,S-Equol (**14**)	138946	93490	701993

Table S5. Yield [%] determination for the chlorinated flavonoids based on the estimated AUC

Flavonoid	6-Br1 [%]	8-Br1 [%]	Unmodified [%]
Chrysin (**2**)	27.1	3.7	69.2
Luteolin (**3**)	1.0	7.4	91.6
Apigenin (**4**)	2.5	21.1	76.4
Tricin (**5**)	78.8	n.d.	21.2
Acacetin (**6**)	9.9	-	90.1
Daidzein (**1**)	39.7	n.d.	60.3
Genistein (**7**)	Not tested	Not tested	Not tested
Biochanin A (**8**)	3.7	5.1	91.2
Quercetin (**9**)	Not tested	Not tested	Not tested
Morin (**10**)	Not tested	Not tested	Not tested
Fisetin (**11**)	Not tested	Not tested	Not tested
Hesperetin (**12**)	2.4	1.4	96.1
Taxifolin (**13**)	Not tested	Not tested	Not tested
R,S-Equol (**14**)	14.9	10.0	75.1

NMR data

Table S6. ^{13}C-NMR (125 MHz) and ^{1}H-NMR (500 MHz) data of 8-Cl-daidzein and reference date from the literature [5].

8-Cl-daidzein (**6a**)			Lit. 8-Cl-daidzein	
**pos.**	**δC (ppm)**	**δH (ppm, *m*, *J* Hz)**	**δC (ppm)**	**δH (ppm, *m*, *J* Hz)**
**CH-2**	154.7	8.26 (1H, s)	152.9	8.25 (1H, s)
**C-3**	126.3		124.9	
**C-4**	177.9		176.4	
**C-4a**	109.0		107.5	
**CH-5**	126.1	8.00 (1H, d J = 8.9)	124.2	7.97 (1H, d, J = 9.0)
**CH-6**	116.2	7.06 (1H, d J = 8.9)	115.9	7.01 (1H, d, J = 9.0)
**C-7**	160.9		161.9	
**C-8**	119.1		116.2	
**C-8a**	155.5		154.2	
**C-1'**	124.0		122.6	
**CH-2'/6'**	131.6	7.37-7.41 (2H, m)	130.1	7.42-7.40 (2H, m)
**CH-3'/5'**	116.4	6.84-6.87 (2H, m)	114.8	6.88-6.86 (2H, m)
**C-4'**	159.1		157.4	

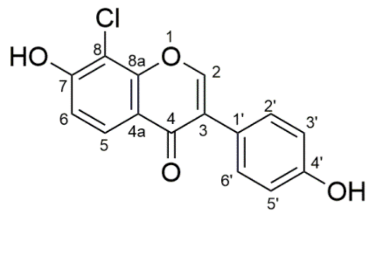

Figure S19. Structure of 8-Cl-daidzein.

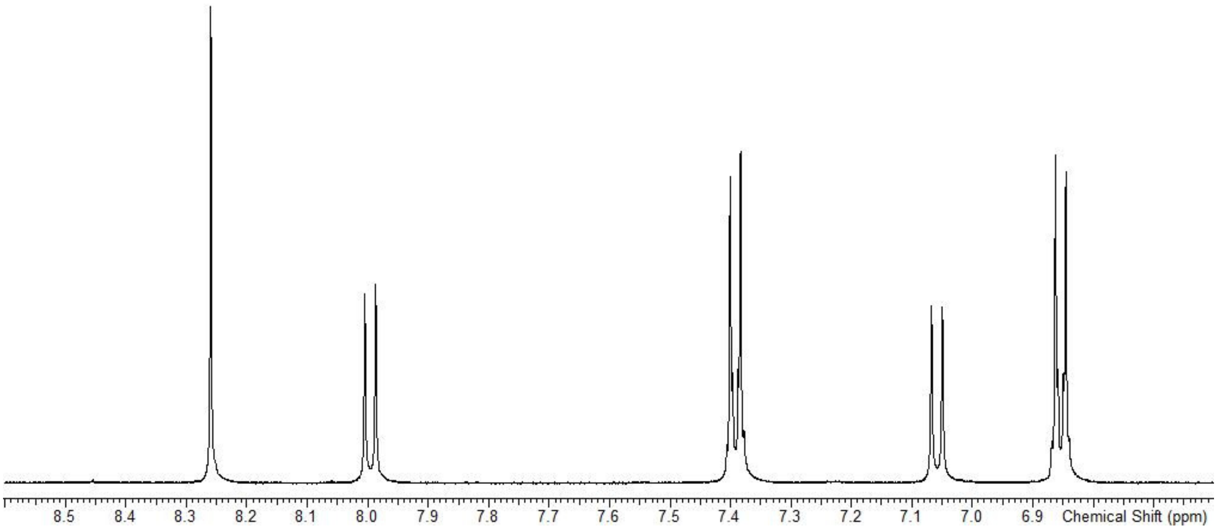

Figure S20. ^{1}H-NMR spectrum of 8-Cl-daidzein (DMSO-d_6, 500 MHz).

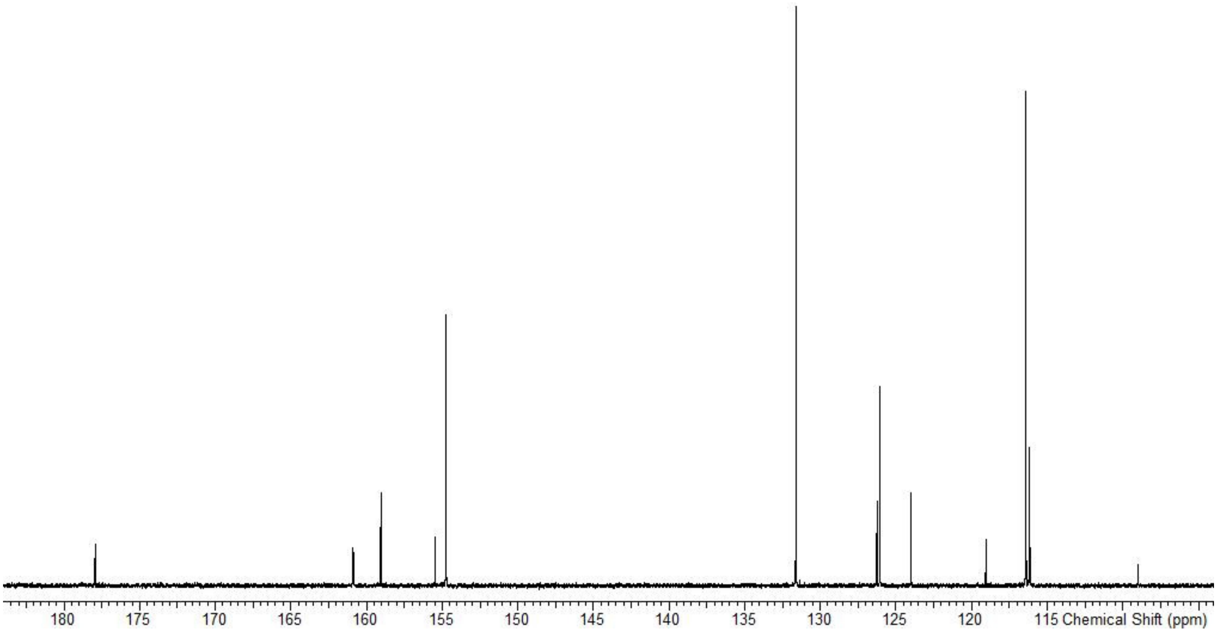

Figure S21. ^{13}C-NMR spectrum of 8-Cl-daidzein (DMSO-d_6, 125 MHz).

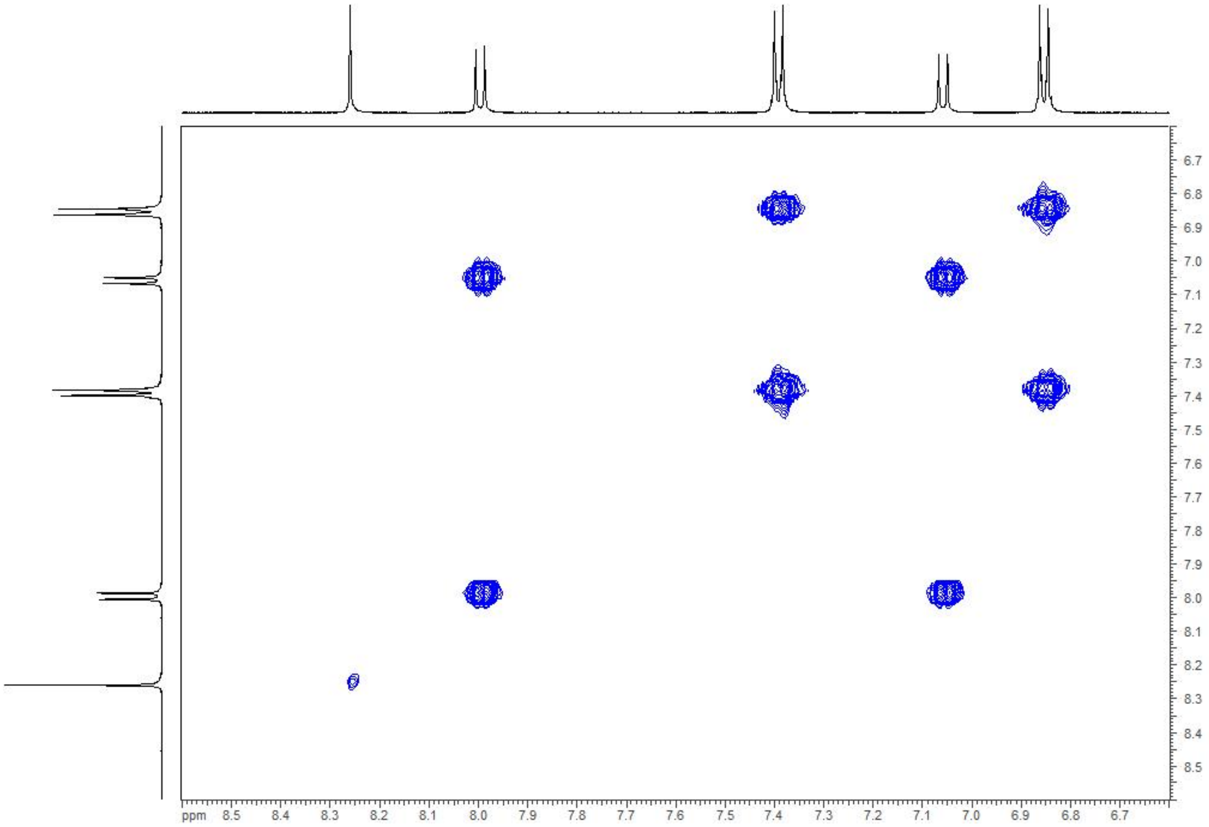

Figure S22. COSY spectrum of 8-Cl-daizein (DMSO-d6, 500 MHz).

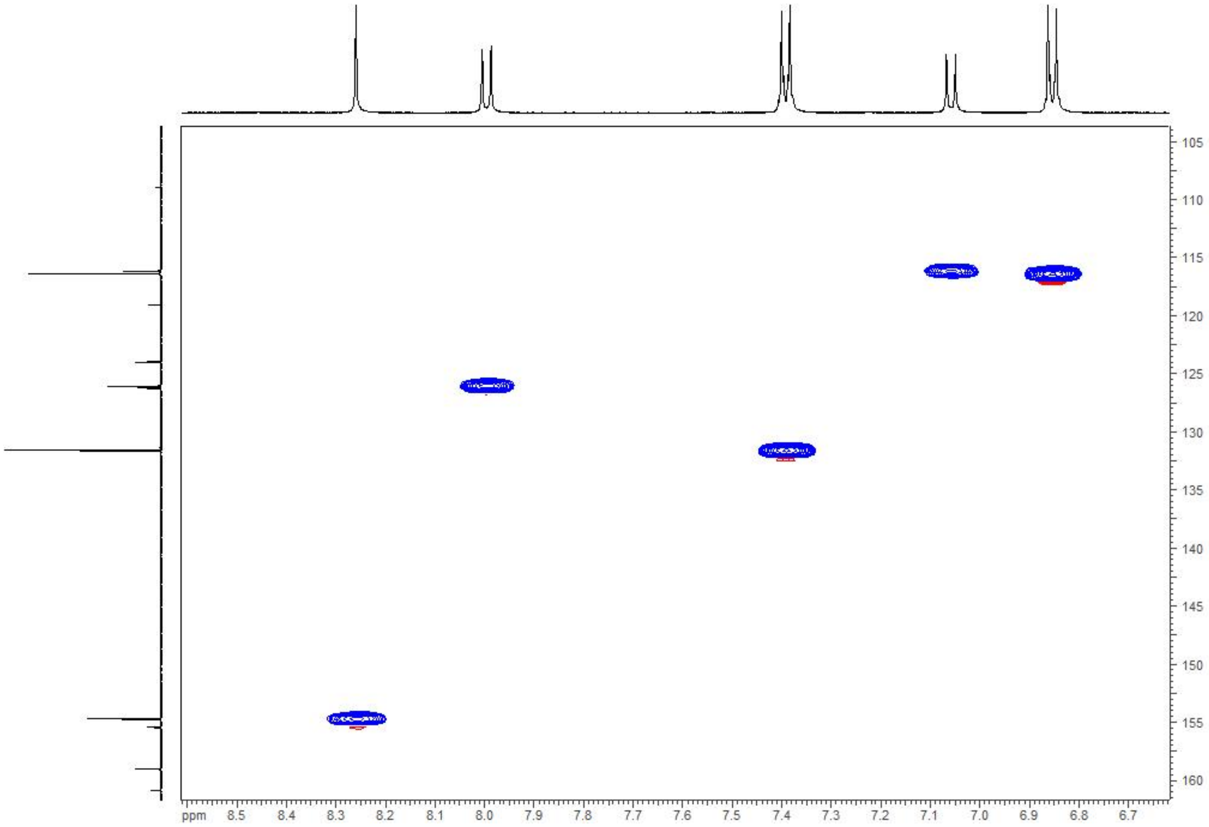

Figure S23. HSQC spectrum of 8-Cl-daidzein (DMSO-d6, 500 MHz).

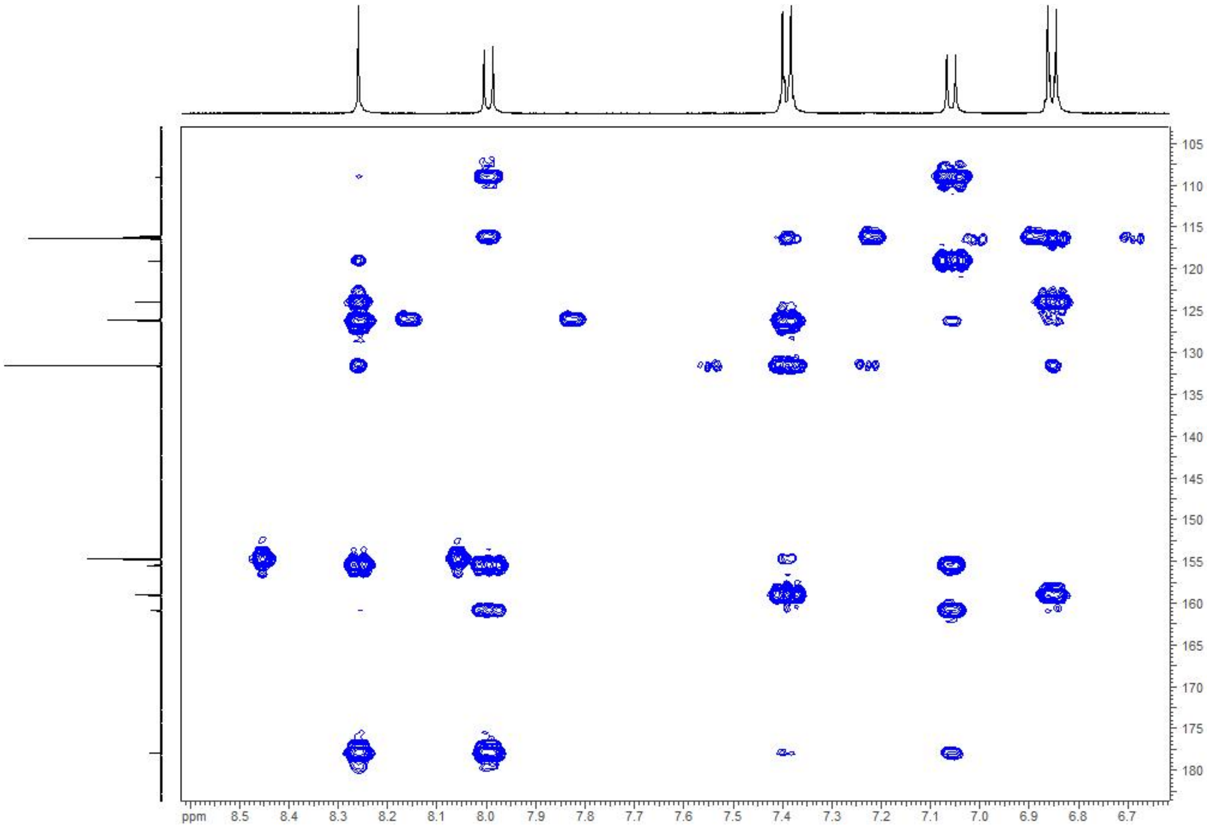

Figure S24. HMBC spectrum of 8-Cl-daidzein (DMSO-d6, 500 MHz).

Table S7. ^{13}C-NMR (125 MHz) and ^{1}H-NMR (500 MHz) data of 6-Cl-chrysin **2a** and the minor side product 8-Cl-chrysin **2b** including their associated HOSE (Hierarchical Organisation of Spherical Environments) code predictions from ACD.

	6-Cl-chrysin (**2a**)		HOSE 6-Cl-chrysin		8-Cl-chrysin (**2b**)		HOSE 8-Cl-chrysin	
**pos.**	**δ_C (ppm)**	**δH (ppm, *m*, *J* Hz)**	**δ_C (ppm)**	**δH (ppm, *m*, *J* Hz)**	**δ_C (ppm)**	**δH (ppm, *m*, *J* Hz)**	**δ_C (ppm)**	**δH (ppm, *m*, *J* Hz)**
**C-2**	163.8		164.4		163.1		163.3	
**CH-3**	105.3	7.04 (1H, *s*)	105.5	6.51(1H, *s*)	n.a**	7.09 (1H, ovl.)	104.9	6.80 (1H, *s*)
**C-4**	181.9		180.9		182.0		180.3	
**C-5**	157.2		155.8		160.8		161.4	
**C/CH-6**	103.4		103.6		n.a**	6.41 (1H, ovl.)	100.0	6.61 (1H, *s*)
**C-7**	160.9		158.1		161.2		160.1	
**C/CH-8**	94.8	6.72 (1H, *s*)	95.6	6.70 (1H, *s*)	98.3		100.5	
**C-9**	155.5		157.1		n.a**		153.5	
**C-10**	104.1		102.1		104.7		105.0	
**C-1'**	130.9		131.1		n.a**		131.1	
**CH-2'/6'**	126.9	8.08 (2H, *d*, *J* = 7.3)	126.5	7.94 (2H, *d*, *J* = 7.2)	n.a**	ovl.*	126.0	7.91 (2H, *d*, *J* = 7.2)
**CH-3'/5'**	129.6	7.58 (2H, *t*, *J* = 7.3)	129.2	7.53, (2H, *t*, *J* = 7.3)	n.a**	ovl.*	129.1	7.49 (2H, *t*, *J* = 7.3)
**CH-4'**	132.6	7.62 (1H, *t*, *J* = 7.3)	132.0	7.55 (1H, *t*, *J* = 7.3)	130.9	ovl.*	131.7	7.51 (1H, *t*, *J* = 7.3)

* overlapping signals ** not available

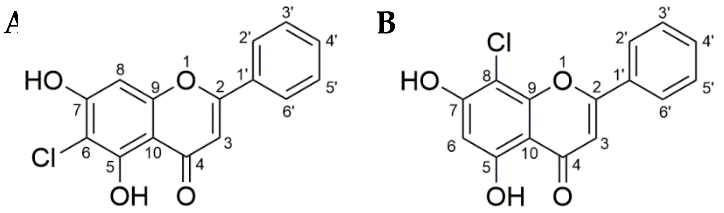

Figure S25. Structure of 6-Cl-chrysin (**A**) and 8-Cl-chrysin (**B**).

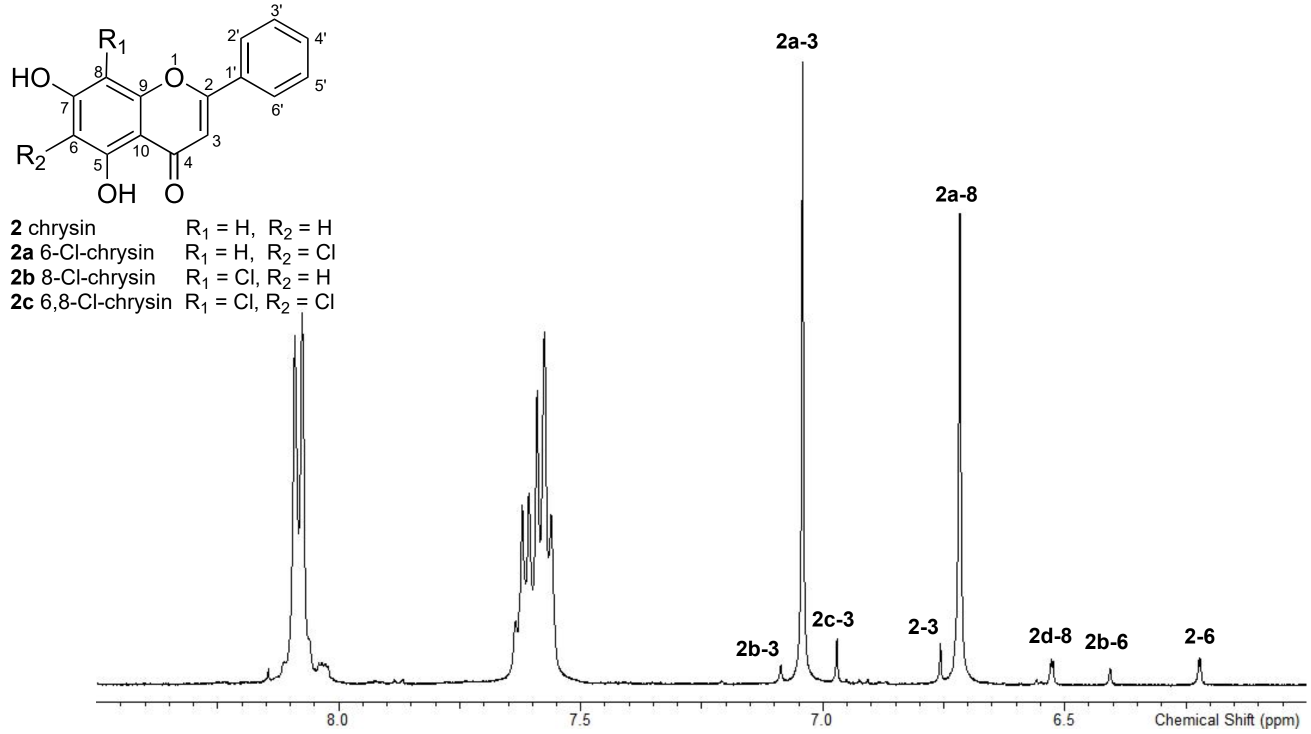

Figure S26. ¹H-NMR spectrum showing the compounds A-D and the signals of protons 3, 6 and 8 of **2a**, **2b**, **2c** and the precursor **2** (DMSO-d6, 500 MHz).

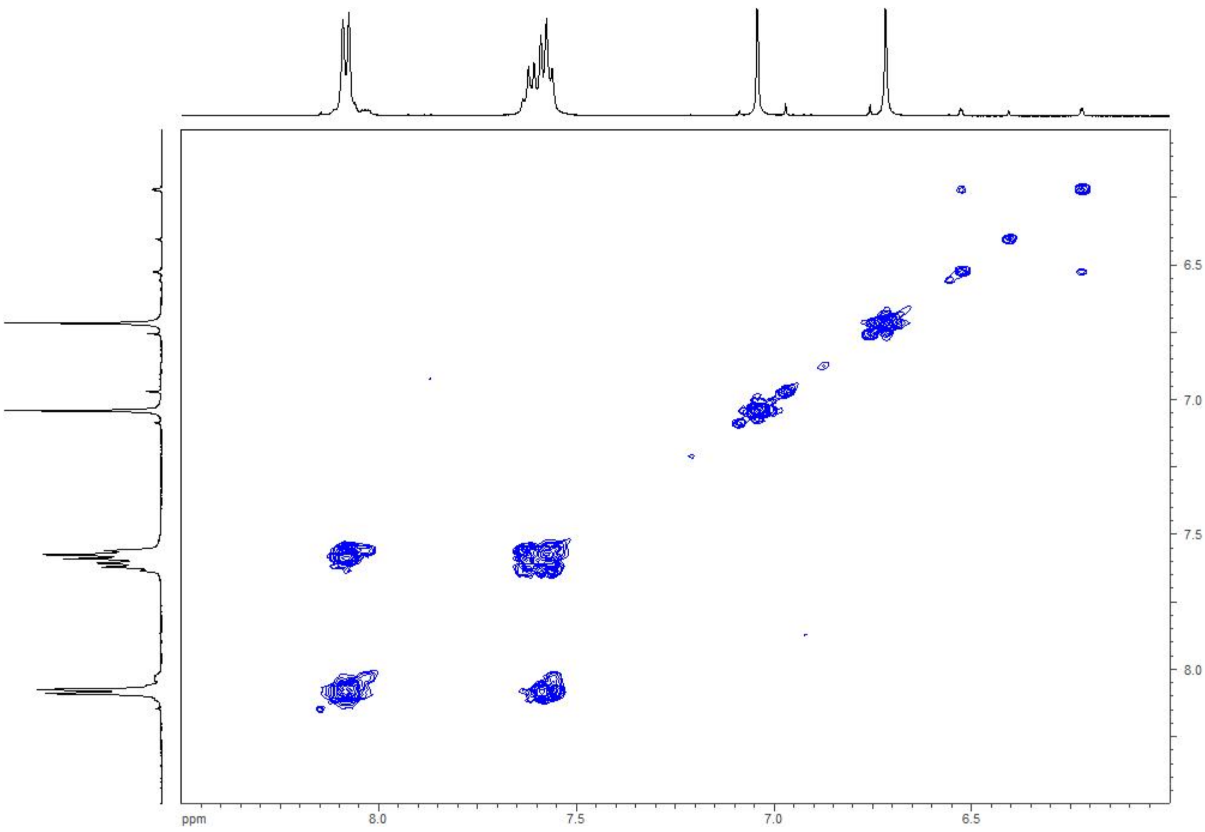

Figure S27. COSY spectrum of 6-Cl-chrysin including side products (DMSO-d_6, 500 MHz).

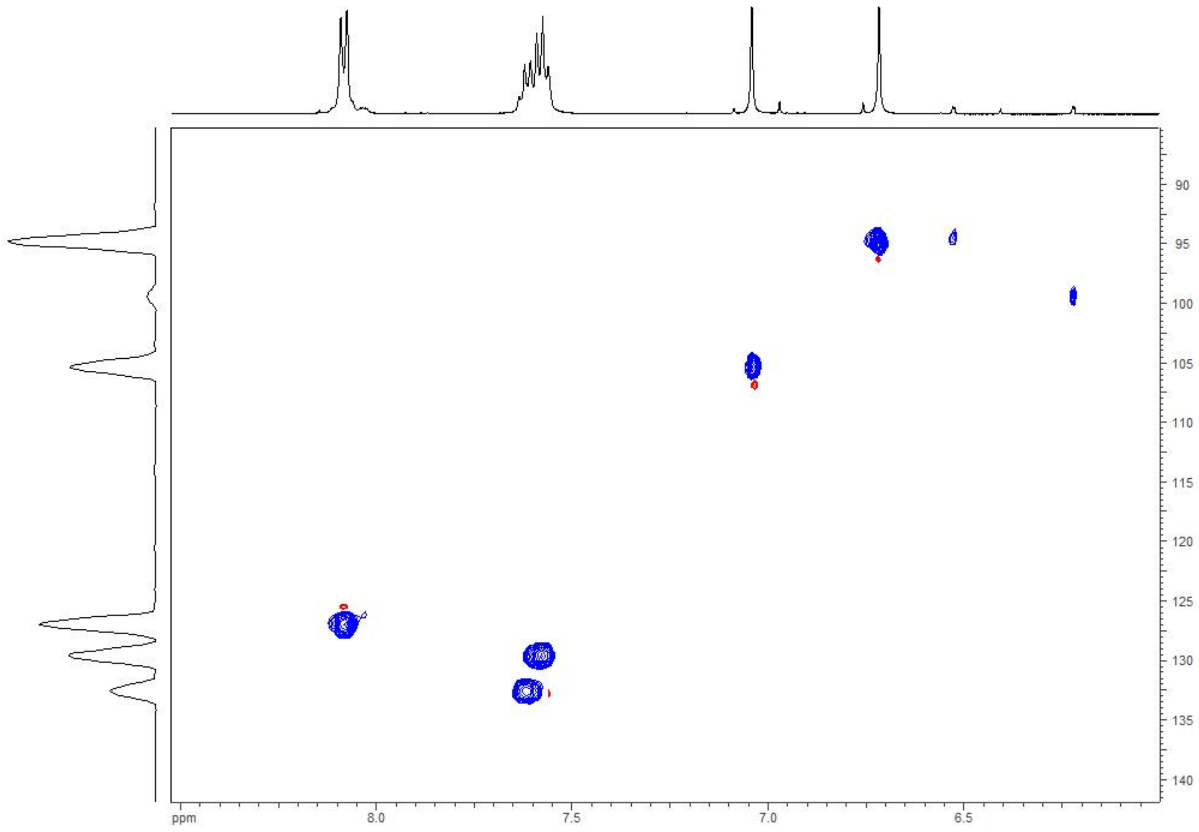

Figure S28. Edited-HSQC spectrum of 6-Cl-chrysin including side products (DMSO-d₆, 500 MHz).

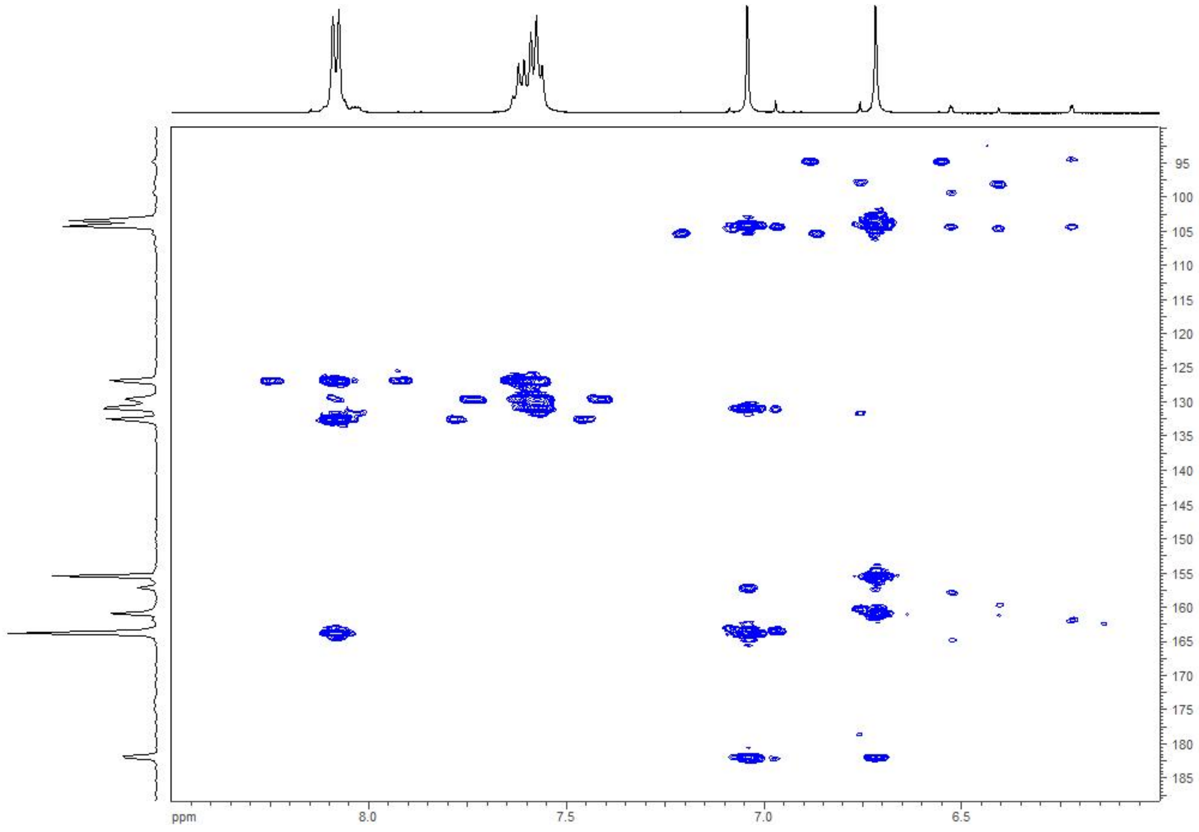

Figure S29. HMBC spectrum of 6-Cl-chrysin including side products (DMSO-d_6, 500 MHz).

Table S8. ^{13}C-NMR (125 MHz) and ^{1}H-NMR (500 MHz) data of 6,8-Cl-chrysin **2c** including the associated HOSE (Hierarchical Organisation of Spherical Environments) code predictions from ACD.

	6,8-Cl-chrysin (**2c**)		HOSE 6,8-Cl-chrysin	
**pos.**	**δC (ppm)**	**δH (ppm, *m*, *J* Hz)**	**δC (ppm)**	**δH (ppm, *m*, *J* Hz)**
**C-2**	161.626		163.81	
**CH-3**	104.655	6.965(1H, ovl.*)	106.41	6.83 (1H, *s*)
**C-4**	180.003		179.59	
**C-5**	155.015		155.92	
**C-6**	105.397		105.84	
**C-7**	155.018		155.45	
**C-8**	99.594		100.13	
**C-9**	151.113		152.42	
**C-10**	100.814		101.61	
**C-1'**	130.816		131.07	
**CH-2'/6'**	126.119	8.068 (2H, ovl.*)	126.00	7.91 (2H, *d*, *J* = 7.2)
**CH-3'/5'**	129.246	7.575 (2H, ovl.*)	129.12	7.49 (2H, *d*, *J* = 7.3)
**CH-4'**	131.928	7.598 (1H, ovl.*)	131.71	7.51 (1H, *d*, *J* = 7.3)

* overlapping signals

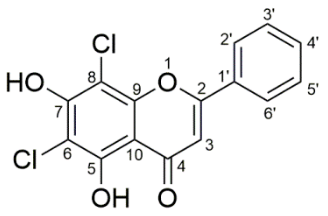

Figure S30. Structure of 6,8-Cl-chrysin.

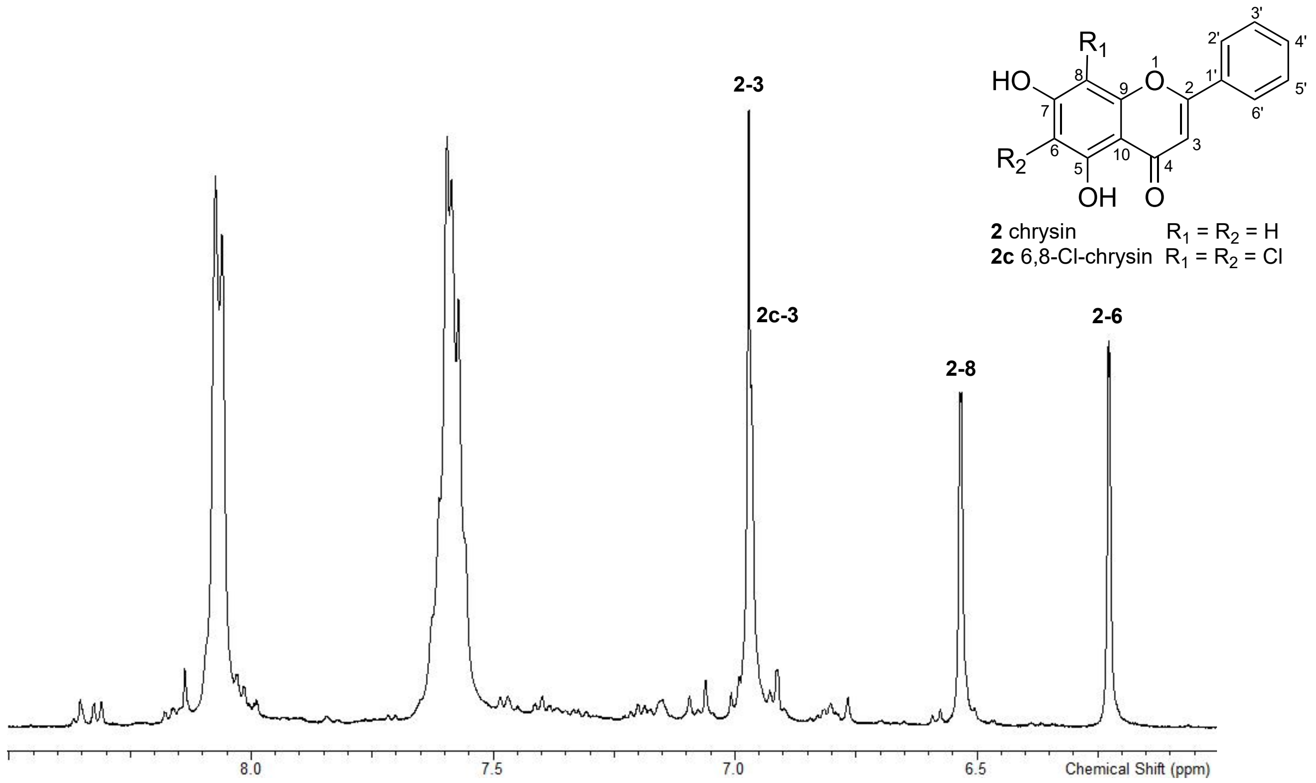

Figure S31. ^{1}H-NMR spectrum showing the compounds 6,8-Cl-chrysin (**2c**) and the precursor chrysin (**2**) including the signals of their protons 3, 6 and 8(DMSO-d6, 500 MHz).

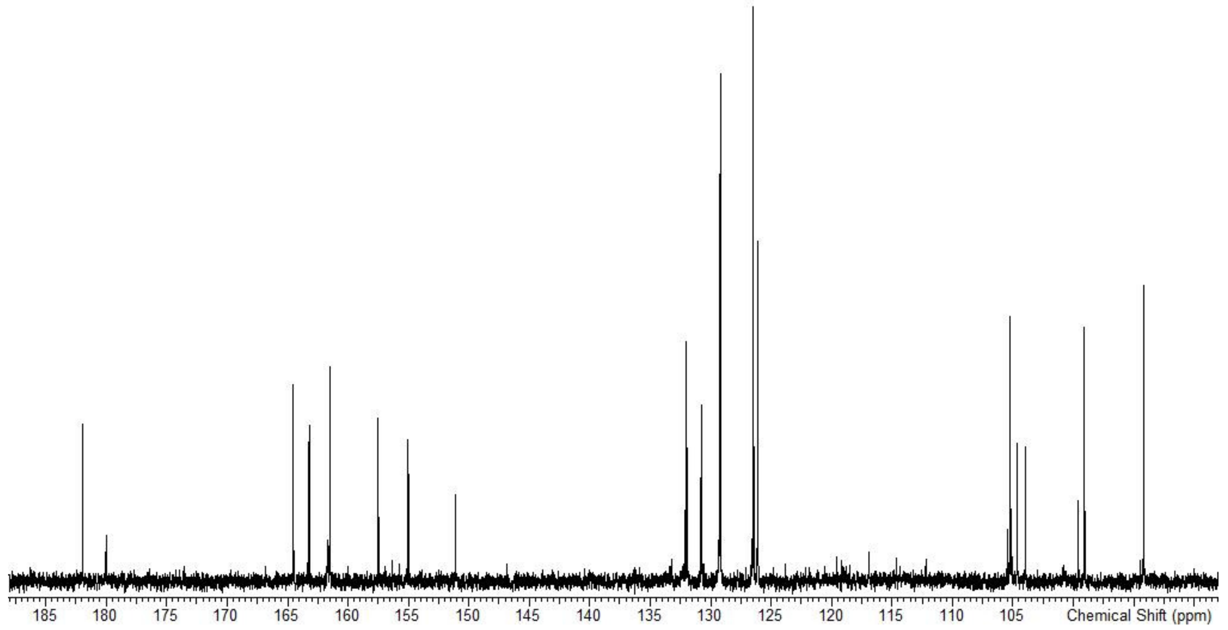

Figure S32. ^{13}C-NMR spectrum of 6,8-Cl-chrysin and the precursor chrysin (DMSO-d6, 125 MHz).

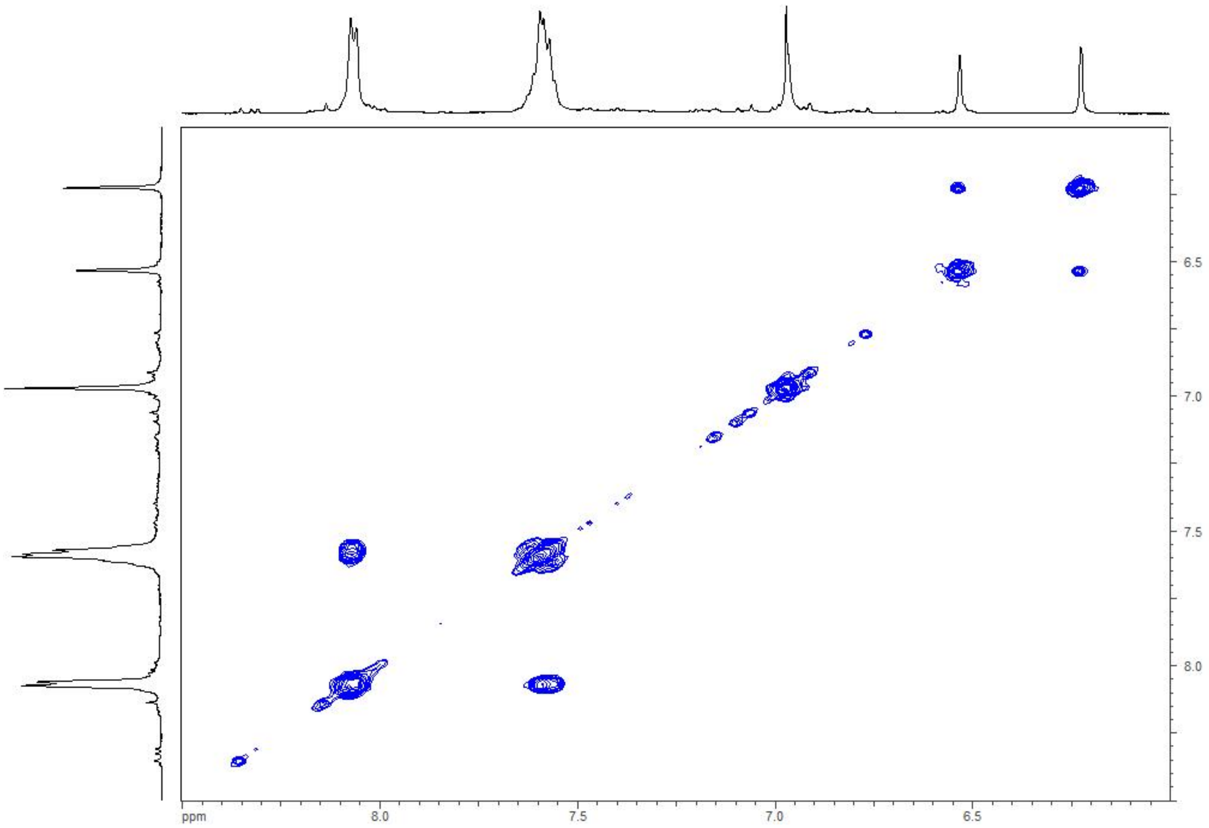

Figure S33. COSY spectrum of 6,8-Cl-chrysin and the precursor chrysin (DMSO-d6, 500 MHz).

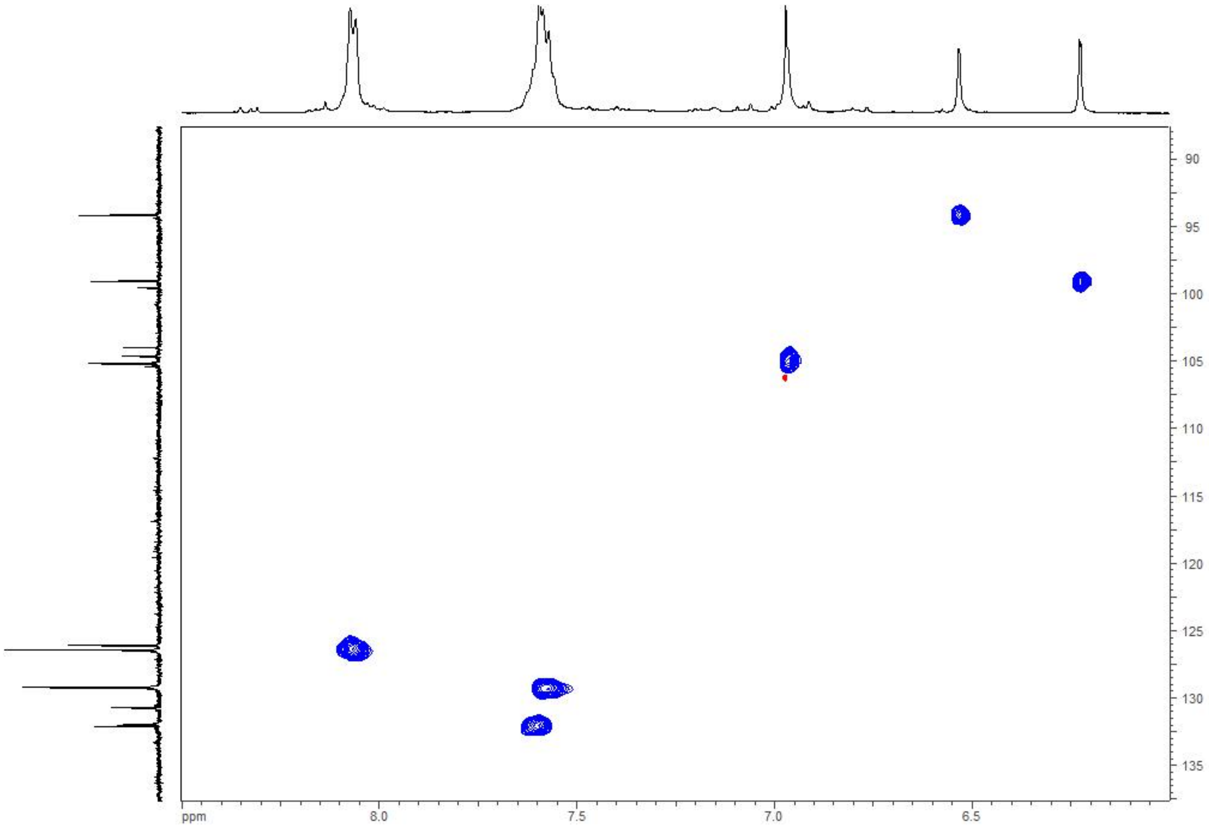

Figure S34. Edited HSQC spectrum of 6,8-Cl-chrysin and the precursor chrysin (DMSO-d_6, 500 MHz).

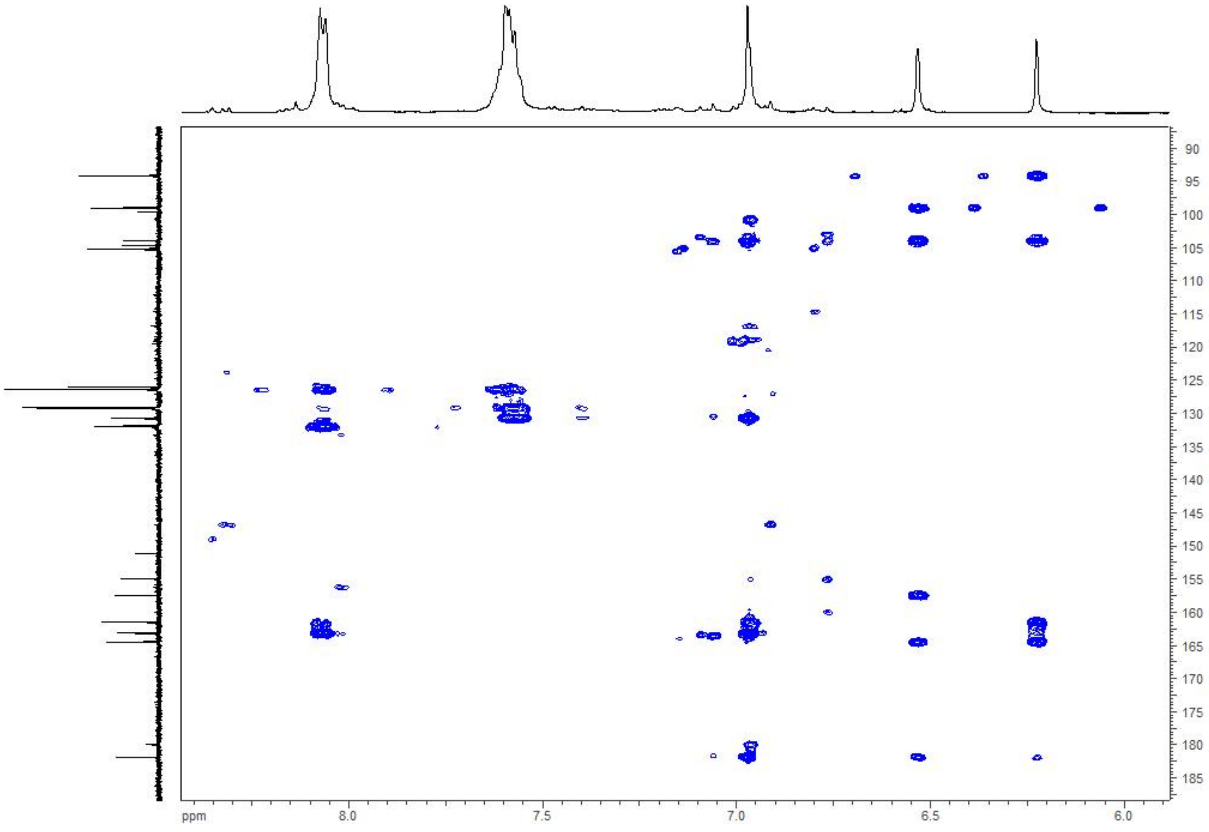

Figure S35. HMBC spectrum of 6,8-Cl-chrysin and the precursor chrysin (DMSO-d6, 500 MHz).

References

1. Myronovskyi, M.; Rosenkränzer, B.; Nadmid, S.; Pujic, P.; Normand, P.; Luzhetskyy, A. Generation of a cluster-free *Streptomyces albus* chassis strains for improved heterologous expression of secondary metabolite clusters. *Metab. Eng*. **2018**, 10.1016/j.ymben.2018.09.004, doi:10.1016/j.ymben.2018.09.004.
2. Flett, F.; Mersinias, V.; Smith, C.P. High Efficiency Intergeneric Conjugal Transfer of Plasmid DNA from *Escherichia Coli* to Methyl DNA-restricting *Streptomycetes*. *Fems Microbiol Lett* **1997**, *155*, 223–229, doi:10.1111/j.1574-6968.1997.tb13882.x.
3. Notredame, C.; Higgins, D.G.; Heringa, J. T-Coffee: A Novel Method for Fast and Accurate Multiple Sequence Alignment. *J. Mol. Biol.* **2000**, 302, 205-217, doi:10.1006/jmbi.2000.4042.
4. Latimer, R.; Podzelinska, K.; Soares, A.; Bhattacharya, A.; Vining, L.C.; Jia, Z.; Zechel, D.L. Expression, Purification and Preliminary Diffraction Studies of CmlS. *Acta Crystallogr Sect F Struct Biology Cryst Commun* **2009**, *65*, 260–263, doi:10.1107/s1744309108043091.
5. Menon, B.R.K.; Brandenburger, E.; Sharif, H.H.; Klemstein, U.; Shepherd, S.A.; Greaney, M.F.; Micklefield, J. RadH: A Versatile Halogenase for Integration into Synthetic Pathways. *Angew Chem-ger Edit* **2017**, *129*, 12003–12007, doi:10.1002/ange.201706342.
